# Supplementary material for: Phase separation of Polo-like kinase 4 by autoactivation and clustering drives centriole biogenesis
Source: Nat Commun. 2019 Oct 31;10:4959. doi: 10.1038/s41467-019-12619-2 (PMC6823436; doi:10.1038/s41467-019-12619-2)
Supplement: Supplementary file 1 — Supplementary Information [file 41467_2019_12619_MOESM1_ESM.pdf]

Supplementary Information for

Phase Separation of Plk4 by Autoactivation and Clustering Drives Centriole Biogenesis

Jung-Eun Park, Liang Zhang, Jeong Kyu Bang, Thorkell Andresson, Frank DiMaio, Kyung S. Lee

This file includes:

Supplementary Figures 1–8

Supplementary References

Supplementary Tables 1–4

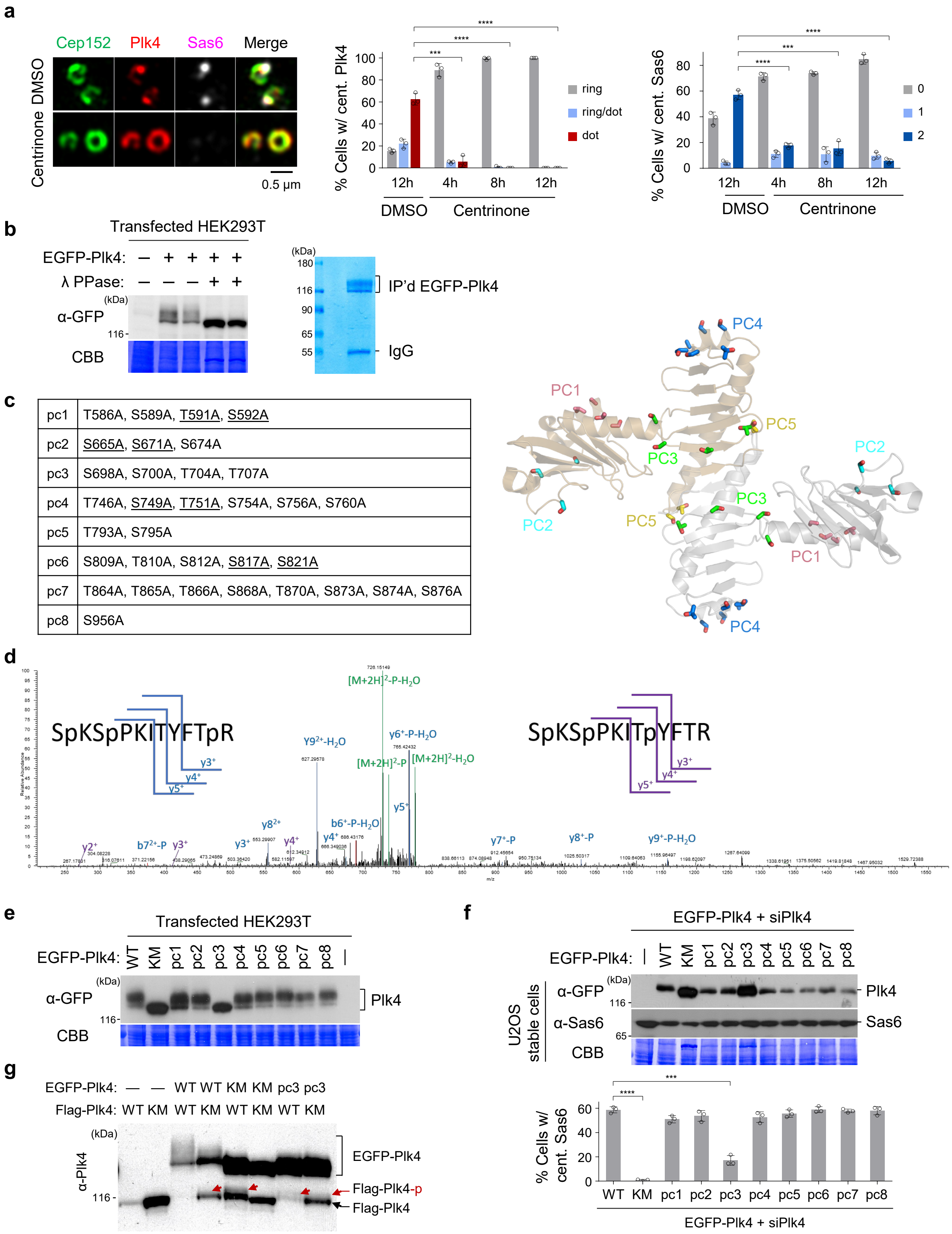

pc1	T586A, S589A, T591A, S592A
pc2	S665A, S671A, S674A
pc3	S698A, S700A, T704A, T707A
pc4	T746A, S749A, T751A, S754A, S756A, S760A
pc5	T793A, S795A
pc6	S809A, T810A, S812A, S817A, S821A
pc7	T864A, T865A, T866A, S868A, T870A, S873A, S874A, S876A
pc8	S956A

Supplementary Figure 1 | Autophosphorylations at the PC3 motif are critical for Plk4's ring-to-dot localization conversion. a, 3D-SIM analysis and quantification of immunostained U2OS cells treated with 200 nM centrinone. Quantified data are shown in mean ± s.d. (n = 3 independent experiments) for each graph. ***, $P < 0.001$; ****, $P < 0.0001$ (unpaired *t*-test). **b,** Immunoprecipitation (IP) and immunoblotting analyses using HEK293T cells transfected with EGFP-Plk4. Where indicated, immunoprecipitated samples were treated with λ phosphatase (PPase). CBB, Coomassie Brilliant Blue–stained membrane. Immunoprecipitated Plk4 was separated by 7.5% sodium dodecyl sulfate–polyacrylamide gel electrophoresis (SDS-PAGE) (right) and excised for MS spectroscopy. **c,** Summary of pc1–8 mutants showing Ser/Thr-phosphorylated residues determined from MS spectroscopy. All phosphoresidues were mutated to Ala. Previously identified phosphoresidues (PhosphoSitePlus; https://www.phosphosite.org/) are underlined. Model (below) depicts the positions of residues in PC1–5. PC6 and PC7 residues present in the unstructured region between PB2 and PB3 are not shown because of no available crystal structure. PC8 in PB3 is also not shown. **d,** MS2 fragment spectra for the Plk4 peptide SKSPKITYFTR (MassIVE Accession #: MSV000084261; Scan number: 4998) was manually annotated, highlighting the y-ions supporting the presence of two forms of the peptide in the spectra triply phosphorylated on either pS698, pS700 and pT704 or pS698, pS700 and pT707. Key y-ions (y2, y3 and y4) supporting phosphorylation on pT707 are highlighted in blue and the y2, y3 and y4 ions supporting phosphorylation on pT704 are highlighted in purple. The common ions to both forms (y1 – y5) exhibit overall higher intensities compared with the specific ions (y2, y3 and y4), further strengthening the presence of two different phosphorylation forms of the peptide. **e,** Immunoblotting analysis of HEK293T cells transfected with the indicated pc mutant constructs separated by 7.5% SDS-PAGE. CBB, Coomassie Brilliant Blue–stained membrane. **f,** Immunoblotting analysis of U2OS cells stably expressing the indicated EGFP-Plk4 constructs depleted of endogenous Plk4 (siPlk4). CBB, Coomassie Brilliant Blue–stained membrane. Note that, likely due to a low level of lentivirus-based Plk4 expression and autophosphorylation, Plk4 mobility shift is not as apparent as that shown in (**e**). Quantification of the cells immunostained with anti-Cep152 and anti-Sas6 antibodies (bottom) to determine the efficiency of Sas6 recruitment to EGFP-Plk4 signals at centrosomes (marked with Cep152). Data are shown in mean ± s.d. (n = 3 independent experiments). ****, $P < 0.0001$ (unpaired *t*-test). **g,** Immunoblotting analysis of HEK293T cells transfected with the indicated constructs. Note that, unlike the catalytically inactive EGFP-Plk4 KM mutant, the EGFP-Plk4 pc3 mutant can transphosphorylate Flag-Plk4 (the slow-migrating Flag-Plk4-p is indicated by red arrows) and induces its degradation nearly as efficiently as EGFP-Plk4 WT {compare lanes 3 (WT) and 7 (pc3 mutant) with lane 5 (catalytically inactive and stable KM)}. However, the increased stability of the EGFP-Plk4 pc3 mutant suggests that PC3 mutations may compromise βTrCP-dependent proteasomal degradation of Plk4.

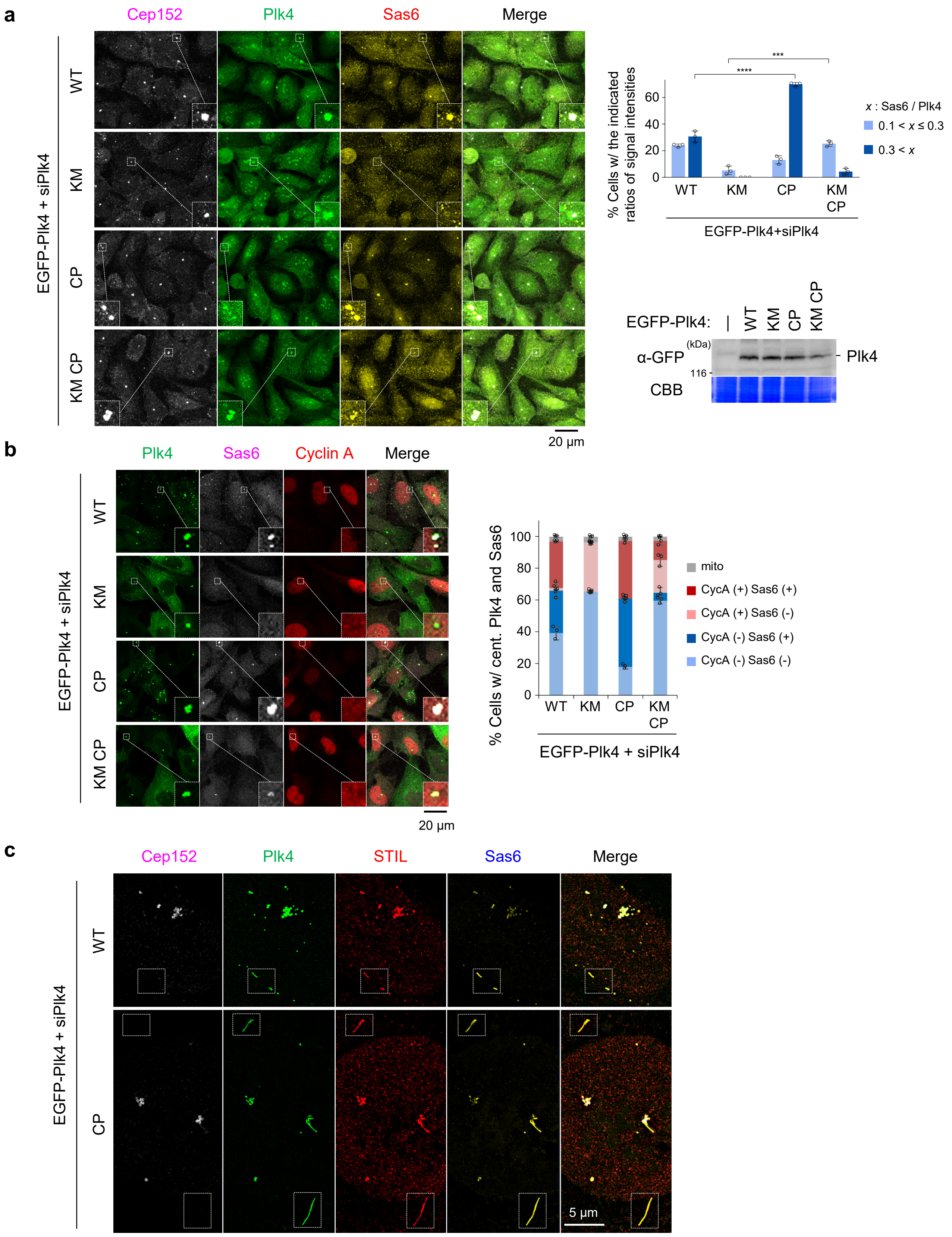
a
Cep152
Plk4
Sas6
Merge
EGFP-Plk4 + siPlk4
WT
KM
CP
KM CP
20 µm
% Cells w/ the indicated ratios of signal intensities
0
20
40
60
****
***
WT
KM
CP
KM CP
EGFP-Plk4+siPlk4
x : Sas6 / Plk4
0.1 < x ≤ 0.3
0.3 < x
EGFP-Plk4:
WT
KM
CP
KM CP
α-GFP
(kDa)
116
Plk4
CBB
b
Plk4
Sas6
Cyclin A
Merge
EGFP-Plk4 + siPlk4
WT
KM
CP
KM CP
20 µm
% Cells w/ cent. Plk4 and Sas6
0
20
40
60
80
100
WT
KM
CP
KM CP
EGFP-Plk4 + siPlk4
mito
CycA (+) Sas6 (+)
CycA (+) Sas6 (-)
CycA (-) Sas6 (+)
CycA (-) Sas6 (-)
c
Cep152
Plk4
STIL
Sas6
Merge
EGFP-Plk4 + siPlk4
WT
CP
5 µm

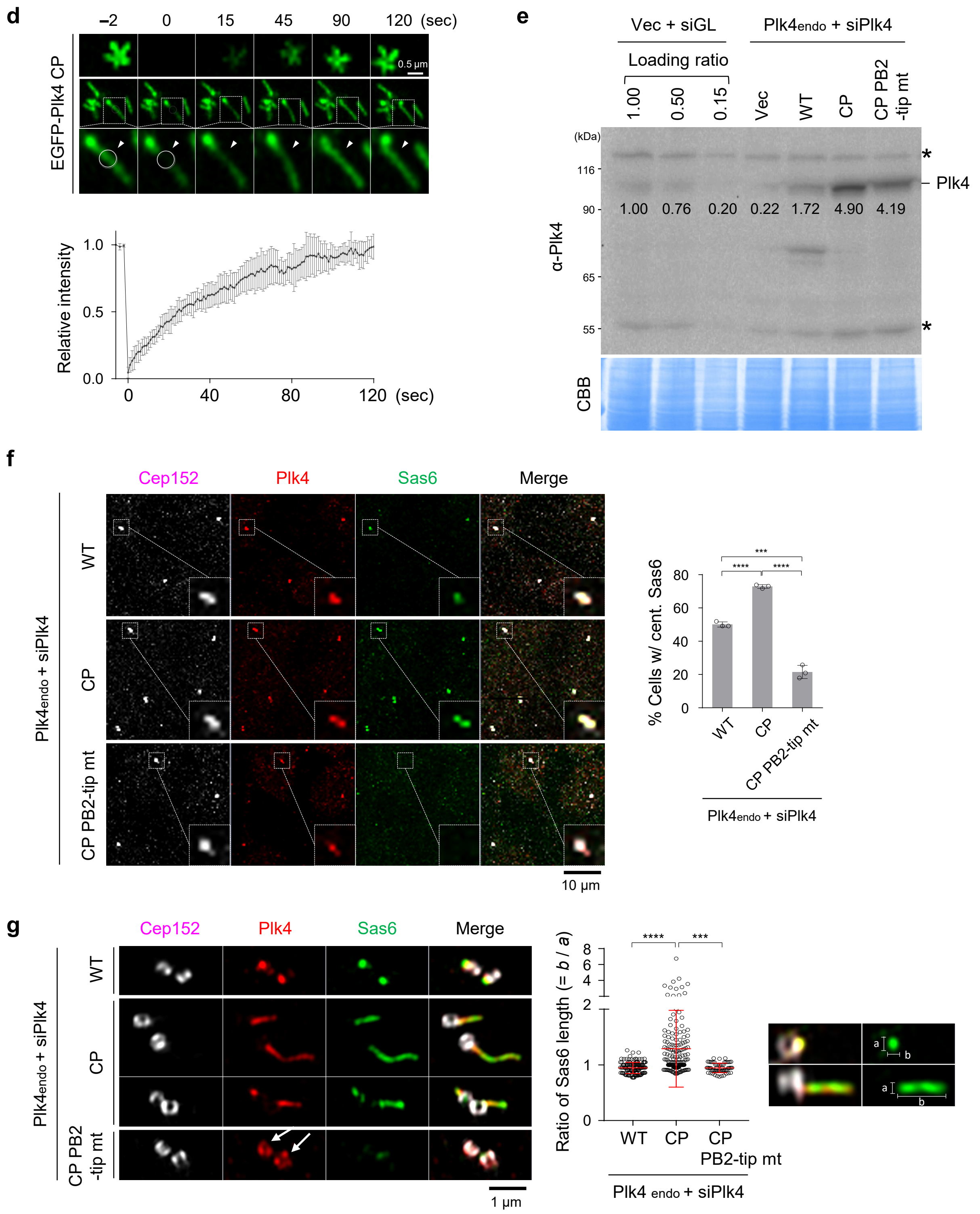

d
−2 0 15 45 90 120 (sec)
EGFP-Plk4 CP
0.5 µm
Relative intensity
1.0
0.5
0.0
0 40 80 120 (sec)
e
Vec + siGL
Plk4endo + siPlk4
Loading ratio
1.00 0.50 0.15
Vec WT CP CP PB2 -tip mt
(kDa)
116
90
65
55
α-Plk4
Plk4
1.00 0.76 0.20 0.22 1.72 4.90 4.19
CBB
f
Cep152 Plk4 Sas6 Merge
WT
CP
CP PB2-tip mt
Plk4endo + siPlk4
10 µm
% Cells w/ cent. Sas6
80 60 40 20 0
***
****
****
WT CP CP PB2-tip mt
Plk4endo + siPlk4
g
Cep152 Plk4 Sas6 Merge
WT
CP
CP PB2 -tip mt
Plk4endo + siPlk4
1 µm
Ratio of Sas6 length (= b / a)
8 6 4 2 1 0
****
***
WT CP CP PB2-tip mt
Plk4 endo + siPlk4
a b
a b

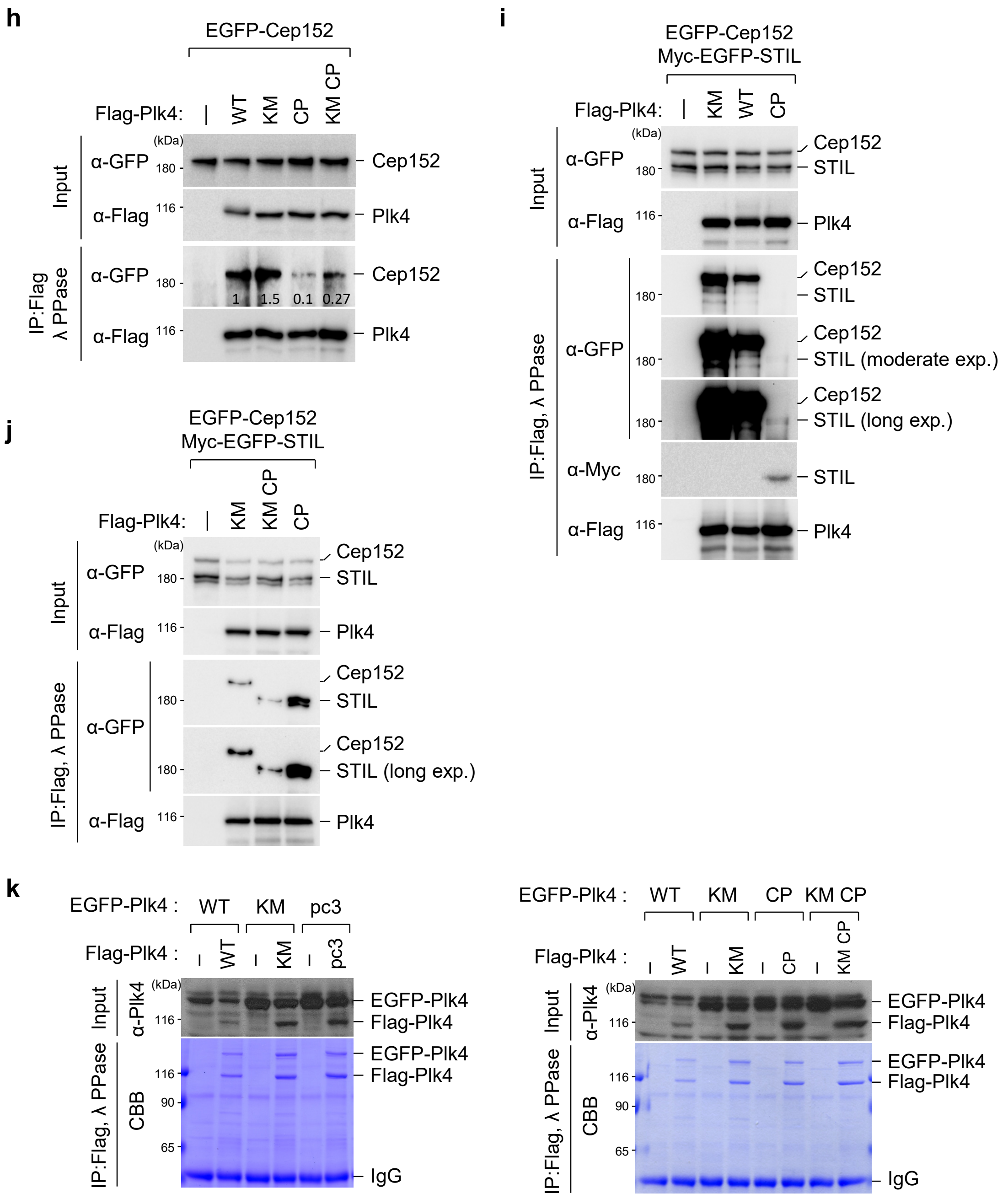

Supplementary Figure 2 | Phospho-mimicking, condensation-proficient mutations at the CPB PC3 motif are sufficient to induce procentriole formation. a, Confocal imaging of immunostained U2OS cells (left) stably expressing various EGFP-Plk4 constructs under Plk4 RNAi (siPlk4) conditions and quantification of Plk4 and Sas6 signal intensities (top right) shown in mean ± s.d. (n = 3 independent experiments). Relative Sas6/Plk4 signal intensities ("*x*") were classified into two groups for detailed comparison. ***, $P < 0.001$; ****, $P < 0.0001$ (unpaired *t*-test). Dotted boxes, areas of image enlargement. Immunoblotting (bottom right) shows the expression level of each construct. CBB, Coomassie Brilliant Blue–stained membrane. **b,** Confocal analysis carried out similarly as in (**a**). Among Cyclin A (CycA)-positive (+) or -negative (–) cells, Sas6 recruited to Plk4-loaded centrosomes was quantified (mean ± s.d., n = 3 independent experiments). **c,** 3D-SIM analysis of immunostained U2OS cells stably expressing the

indicated constructs under Plk4 RNAi (siPlk4) conditions. The dotted boxes indicate the cytosolic (as judged by the absence of the centrosomal Cep152 signal) regions used to generate the images shown in Fig. 2b. **d,** FRAP analyses were carried out after photobleaching either the entire region of an early-stage, star-shape EGFP-Plk4 CP (top) or the indicated region (arrow) of late-stage, elongated EGFP-Plk4 CP (bottom; see the enlarged image) in U2OS cells. Images were taken either every 0.5 seconds (top) or every second (bottom) for the length of 120 seconds. Photobleaching was carried out at 0 seconds (0 s). Relative signal intensities were quantified with both dot-like and elongated EGFP-Plk4 CP signals. Data are shown in mean ± s.d. (n = 7 independent experiments). **e,** Immunoblotting analysis showing the level of Plk4 expression in U2OS cells stably expressing endogenous promoter–controlled Plk4 WT-*sil* or the indicated CP-*sil* or CP PB2-tip mut-*sil* mutant under Plk4 RNAi (siPlk4) conditions. Numbers, relative signal intensities. Asterisks, cross-reacting proteins. CBB, Coomassie Brilliant Blue–stained membrane. **f,** Confocal imaging analysis for the cells in (**e**) after immunostaining with the indicated antibodies. Quantification of images is shown in mean ± s.d. (n = 3 independent experiments). ***, $P < 0.001$; ****, $P < 0.0001$ (unpaired *t*-test). **g,** 3D-SIM analysis for the same samples in (**f**). Note that, unlike Plk4 WT, Plk4 CP exhibited noticeably elongated signals colocalized with Sas6. Arrows indicate ring-like signals observed with the condensation-defective Plk4 CP PB2-tip mutant (see Fig. 6). The ratio of the Sas6 signal length over its width was determined with centriole-associated signals. Quantified data are shown in mean ± s.d. (n = 3 independent experiments). ***, $P < 0.001$; ****, $P < 0.0001$ (unpaired *t*-test). **h,** IP and immunoblotting analyses using HEK293T cells cotransfected with the indicated constructs. Immunoprecipitated samples were then treated with λ phosphatase (PPase) and separated by 8% SDS-PAGE for immunoblotting. Numbers, relative signal intensities. **i,** IP and immunoblotting analyses using a mixture of lysates from HEK293T cells transfected separately with each of the indicated constructs. Samples were analyzed as in (**h**) except that the immunoprecipitates were subjected to anti-Myc immunoblotting to confirm the coprecipitating Myc-EGFP-STIL with Flag-Plk4 CP. Note that, unlike the Cep152–Plk4 interaction [1], Plk4 kinase activity is important for efficient interaction between Plk4 and STIL [2]. Therefore, the weak Plk4–STIL interaction may not account for all the Plk4 kinase activity–dependent Plk4–STIL interaction that may require prior *in vivo* phosphorylation and/or physicochemical change, such as condensation. **j,** IP and immunoblotting analyses were carried out as in (**h**) using HEK293T cells cotransfected with the indicated constructs. Note that the CP mutations even in the catalytically inactive Plk4 KM mutant are sufficient to induce the Plk4-STIL interaction. **k,** IP and immunoblotting analyses using HEK293T cells cotransfected with the indicated constructs. IP samples were then treated with λ phosphatase (PPase) and analyzed as in (**h**). CBB, Coomassie Brilliant Blue–stained membrane.

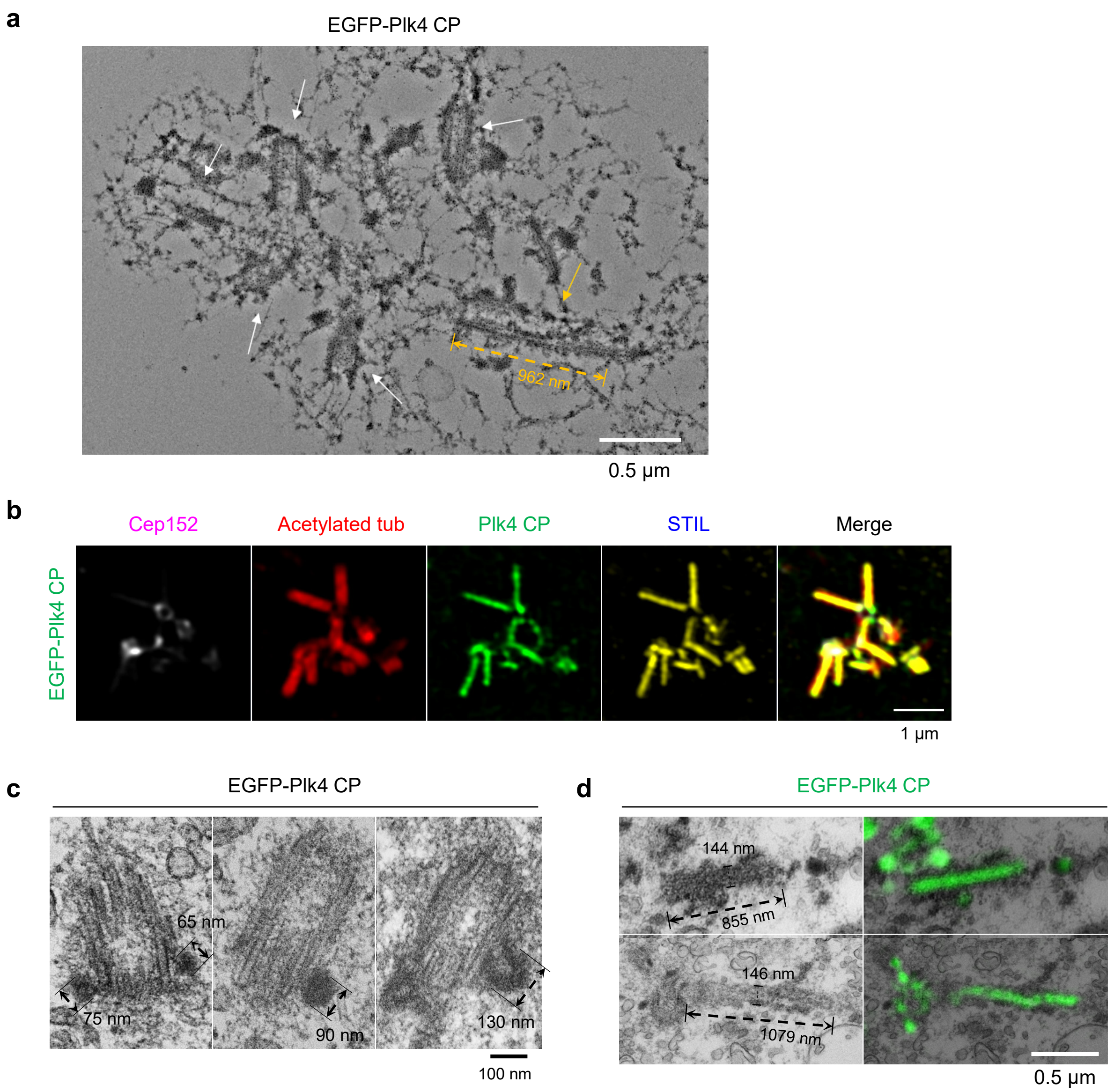

Supplementary Figure 3 | The Plk4 CP mutant effectively generates nanoscale spherical condensates at the pericentriolar region and is capable of inducing an elongated centriole. a, Thin-section transmission electron microscopy (TEM) of U2OS cells stably expressing EGFP-Plk4 CP shows multiple centrioles (white arrows) and an unusually elongated centriole (yellow arrow) with apparent centriolar MT structures. **b,** The same cells used for **a** were immunostained with the indicated antibodies and then imaged by 3D-SIM. Cep152 marks the location of endogenous centrosomes and acetylated tubulin signals indicate that the elongated signals are centrioles **c,** Thin-section TEM of U2OS cells stably expressing EGFP-Plk4 CP reveals spherical Plk4 condensates at the pericentriolar region. The diameters of Plk4 spheres are shown. **d,** Additional images of correlative light and electron microscopy (CLEM) showing elongated procentrioles observed in U2OS cells stably expressing EGFP-Plk4 CP. Also see the images in Fig. 3b. Overlaid images with GFP fluorescence are shown on the right. The dimensions of Plk4 CP-induced, electron-dense matrix-like bodies are indicated.

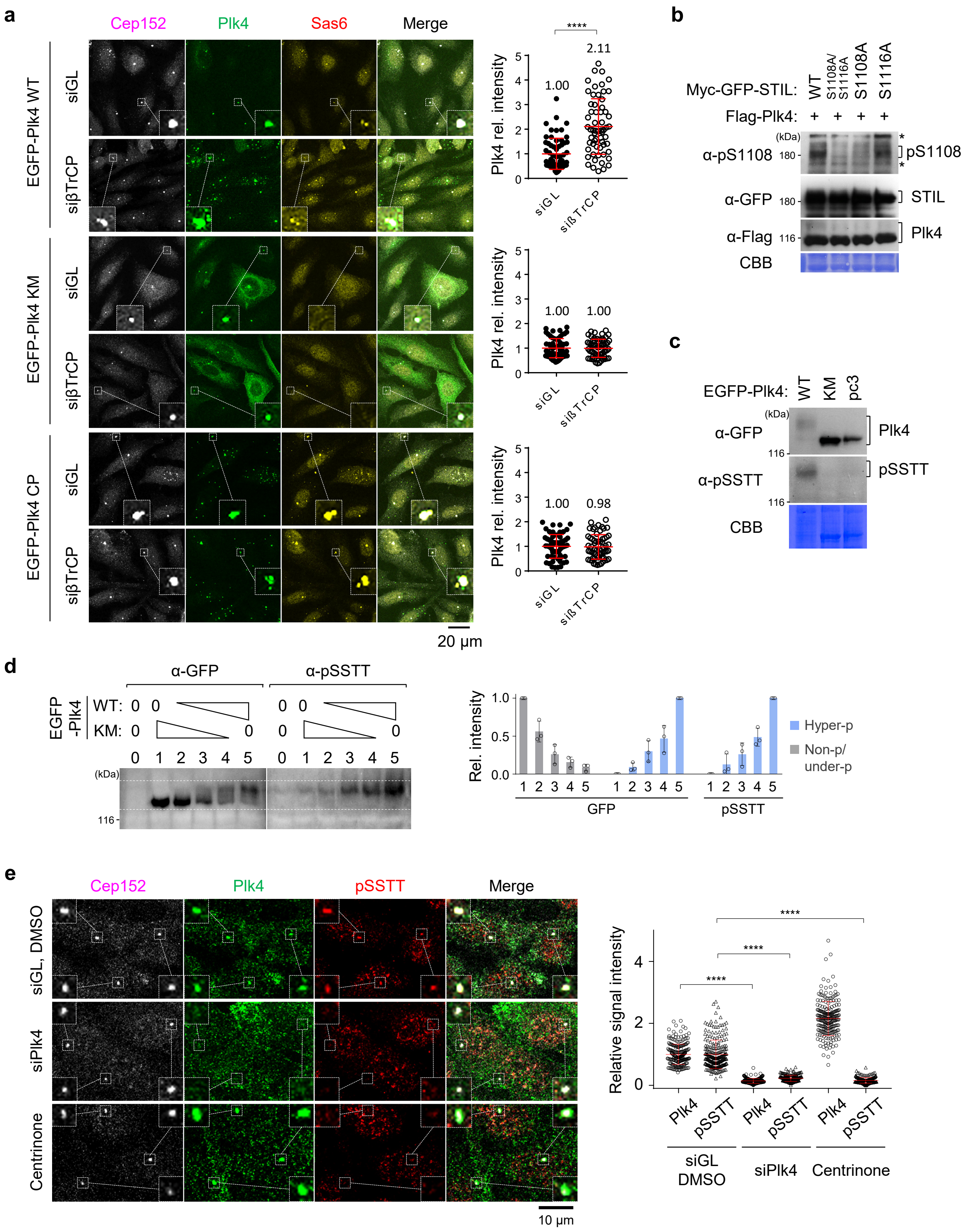

a
Cep152
Plk4
Sas6
Merge
EGFP-Plk4 WT
EGFP-Plk4 KM
EGFP-Plk4 CP
siGL
siβTrCP
Plk4 rel. intensity
****
1.00
2.11
1.00
1.00
1.00
0.98
20 μm
b
Myc-GFP-STIL:
WT
S1108A/S1116A
S1108A
S1116A
Flag-Plk4:
+ + + +
(kDa)
α-pS1108
180
pS1108
α-GFP
STIL
α-Flag
116
Plk4
CBB
c
EGFP-Plk4:
WT
KM
pc3
α-GFP
α-pSSTT
pSSTT
d
α-GFP
α-pSSTT
EGFP-Plk4
WT:
KM:
Rel. intensity
Hyper-p
Non-p/under-p
GFP
pSSTT
e
Cep152
Plk4
pSSTT
Merge
siGL, DMSO
siPlk4
Centrinone
10 μm
Relative signal intensity
siGL DMSO
Centrinone

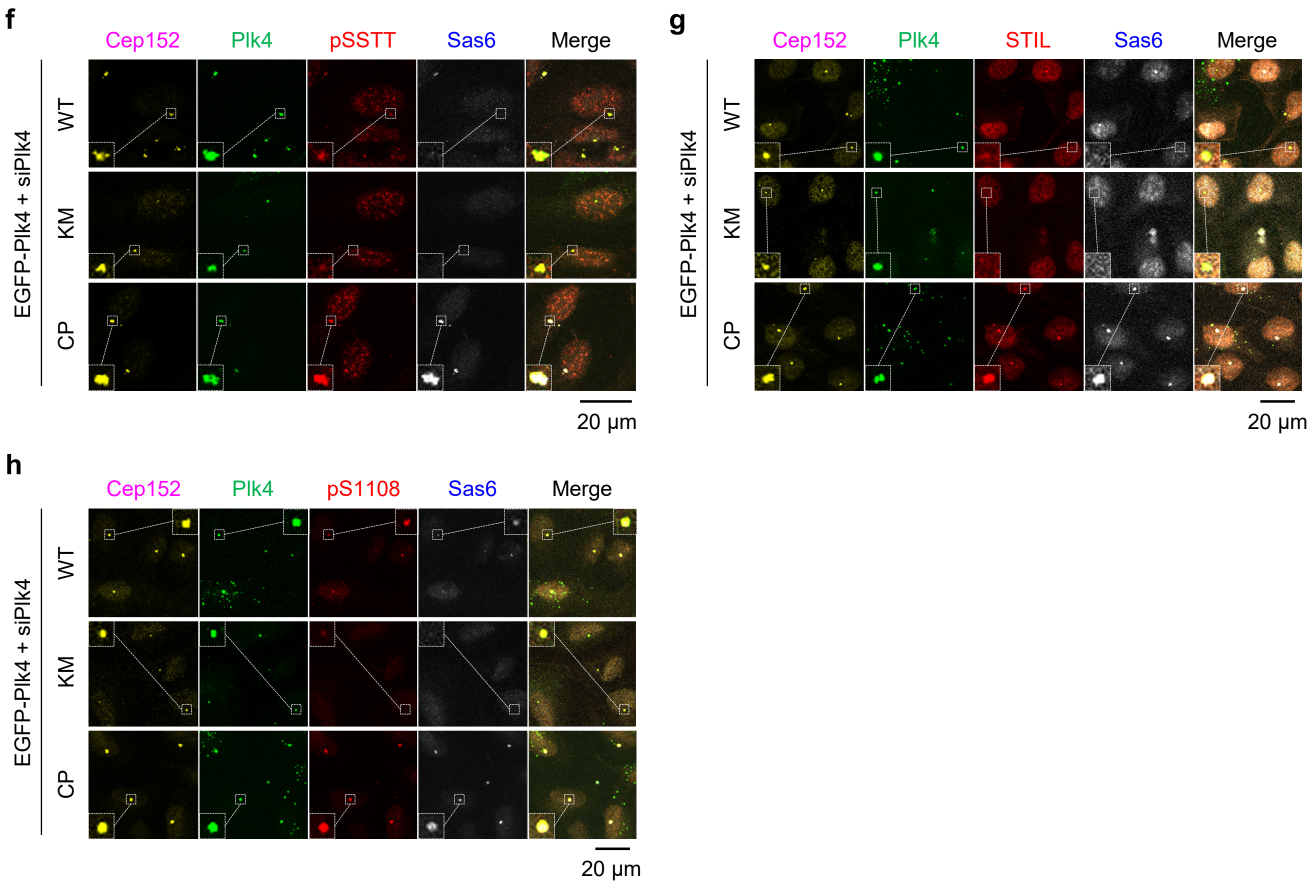

Supplementary Figure 4 | An autophosphorylation-dependent robust generation of the Plk4 pSSTT epitope and the potency of a βTrCP-insensitive Plk4 CP in the recruitment of STIL and Sas6. a, Confocal analysis of U2OS cells stably expressing the indicated EGFP-Plk4 constructs under control luciferase or βTrCP RNAi (siGL or siβTrCP, respectively) conditions. Quantification of relative fluorescence intensities is shown in mean ± s.d. (n = 61/sample from 3 independent experiments). ****, $P < 0.0001$ (unpaired *t*-test). Dotted boxes, areas of enlargement. Numbers, mean values. **b,c,** Immunoblotting analyses of HEK293T cells transfected with the indicated constructs. The S1108 and S1116 residues in (**b**) are two of the Plk4-dependent phosphorylation sites found on the STIL STAN motif [2,3]. The pc3 mutant in (**c**) contains S698A S700A T704A and T707A quadruple mutations. Asterisk, cross-reacting protein. CBB, Coomassie Brilliant Blue–stained membrane. **d,** Immunoblotting analyses of HEK293T cells transfected with varying ratios of EGFP-Plk4 WT or KM (as indicated by the triangular diagrams) and quantification of relative signal intensities for total EGFP-Plk4 (α-GFP) and the Plk4 pSSTT epitope (α-pSSTT). Graph shows an average of three independent experiments. Hyper-p, slow-migrating hyperphosphorylated forms. Non-p/under-p, fast migrating, non-phosphorylated/under-phosphorylated forms. Dotted lines were drawn to indicate corresponding positions in each gel. **e,** Confocal analysis of U2OS cells treated with siRNA (siGL or siPlk4) or 200 nM centrinone for 12 h. The resulting cells were immunostained with anti-Cep152, anti-Plk4, or Alexa Fluor 594–conjugated anti-Plk4 pSSTT antibodies. Dotted boxes, areas of enlargement. Quantification of relative fluorescence intensities is shown in mean ± s.d. (n > 211/sample from 3 independent experiments). ****, $P < 0.0001$ (unpaired *t*-test). **f–h,** Confocal analysis of immunostained U2OS cells stably expressing various EGFP-Plk4 constructs. Dotted boxes, areas of enlargement. Quantification was conducted to generate Fig. 4d.

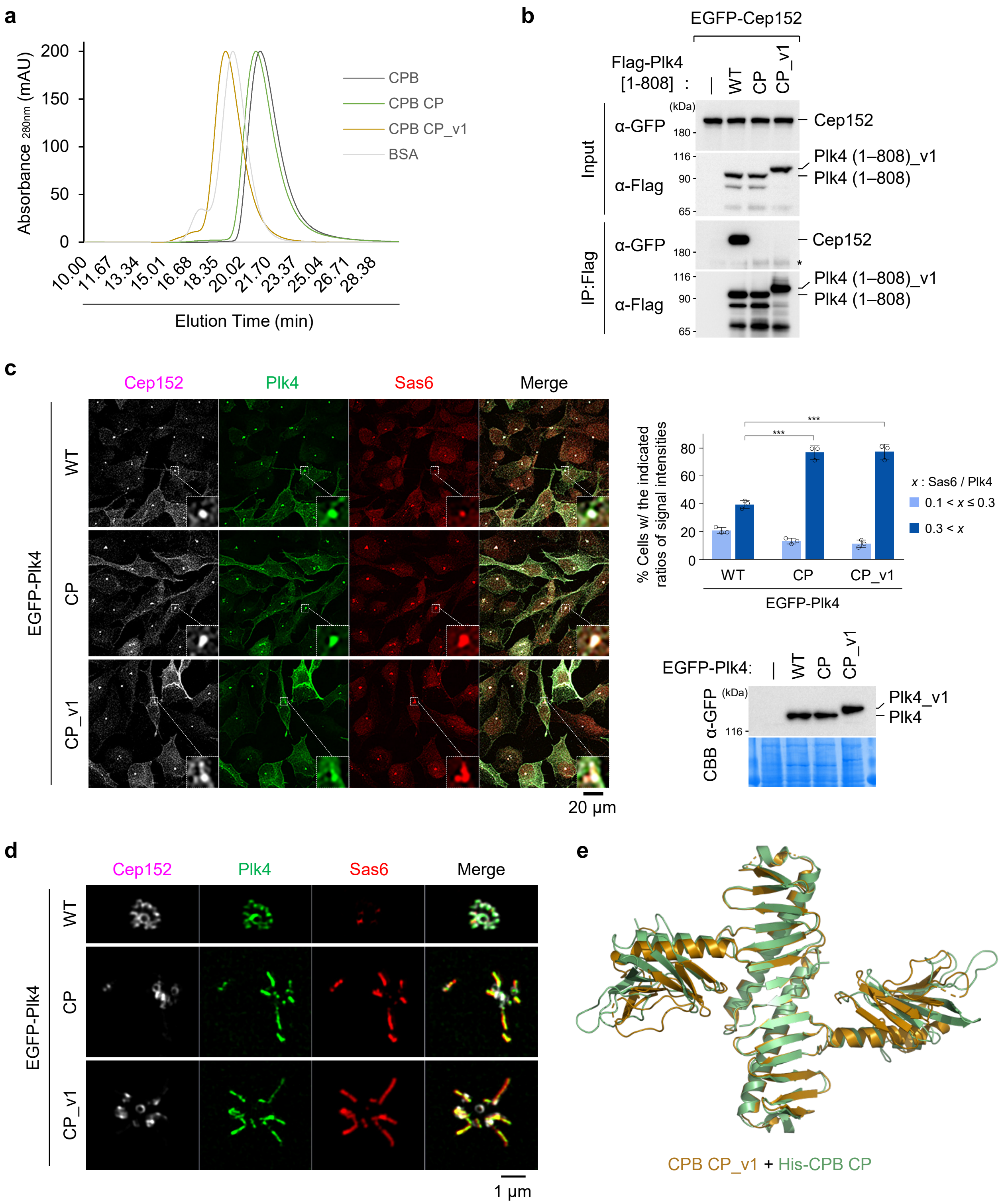

a
Absorbance 280nm (mAU)
200
150
100
50
0
10.00 11.67 13.34 15.01 16.68 18.35 20.02 21.70 23.37 25.04 26.71 28.38
Elution Time (min)
CPB
CPB CP
CPB CP_v1
BSA
b
EGFP-Cep152
Flag-Plk4 [1-808] :
WT
CP
CP_v1
Input
IP:Flag
α-GFP
α-Flag
(kDa)
180
116
90
65
Cep152
Plk4 (1–808)_v1
Plk4 (1–808)
c
Cep152
Plk4
Sas6
Merge
EGFP-Plk4
WT
CP
CP_v1
20 μm
% Cells w/ the indicated ratios of signal intensities
***
***
x : Sas6 / Plk4
0.1 < x ≤ 0.3
0.3 < x
EGFP-Plk4:
α-GFP
CBB
Plk4_v1
Plk4
d
1 μm
e
CPB CP_v1 + His-CPB CP

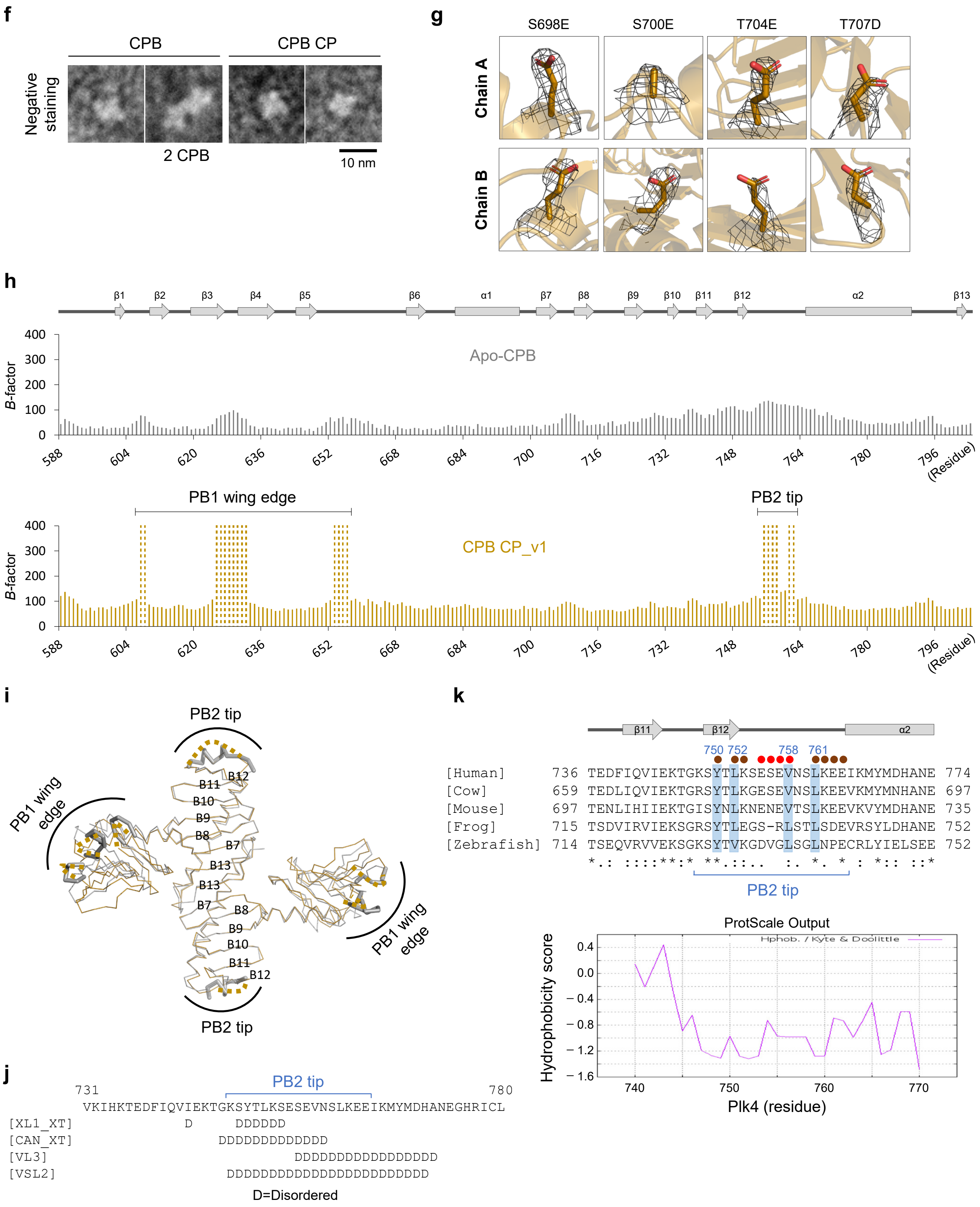
f
CPB
CPB CP
Negative staining
2 CPB
10 nm
g
S698E
S700E
T704E
T707D
Chain A
Chain B
h
β1
β2
β3
β4
β5
β6
α1
β7
β8
β9
β10
β11
β12
α2
β13
Apo-CPB
B-factor
(Residue)
PB1 wing edge
PB2 tip
CPB CP_v1
i
PB2 tip
PB1 wing edge
B11
B12
B10
B9
B8
B7
B13
k
β11
β12
α2
750 752
758
761
[Human] 736 TEDFIQVIEKTGKSYTLKSESEVNSLKEEIKMYMDHANE 774
[Cow] 659 TEDLIQVIEKTGRSYTLKGESEVNSLKEEVKMYMNHANE 697
[Mouse] 697 TENLIHIIEKTGISYNLKNENEVTSLKEEVKVYMDHANE 735
[Frog] 715 TSDVIRVIEKSGRSYTLEGS-RLSTLSDEVRSYLDHANE 752
[Zebrafish] 714 TSEQVRVVEKSGKSYTVKGDVGLSGLNPECRLYIELSEE 752
ProtScale Output
Hphob. / Kyte & Doolittle
Hydrophobicity score
Plk4 (residue)
j
731
780
VKIHKTEDFIQVIEKTGKSYTLKSESEVNSLKEEIKMYMDHANEGHRICL
[XL1_XT] D DDDDDD
[CAN_XT] DDDDDDDDDDDDD
[VL3] DDDDDDDDDDDDDDDDD
[VSL2] DDDDDDDDDDDDDDDDDDDDDDDD
D=Disordered

Supplementary Figure 5 | Increased flexibility in the PB1 wing edge and PB2-tip regions induced by the condensation-proficient CP mutations. a, Size-exclusion chromatography elution profiles of the homodimeric CPB, CPB CP, or CPB CP_v1 complexes. A 66-kDa bovine serum albumin (BSA) serves as a control. **b,** IP and immunoblotting analyses using HEK293T cells cotransfected with the EGFP-Cep152 full-length and Flag-Plk4 (1–808), Flag-Plk4 (1–808) CP, or Flag-Plk4 (1–808)_v1. **c,** Analyses of U2OS cells stably expressing EGFP-Plk4 WT, EGFP-Plk4 CP, or EGFP-Plk4 CP_v1 by immunostaining and confocal microscopy (left and top right) and immunoblotting (bottom right). Dotted boxes, areas of image enlargement. Relative Sas6/Plk4 signal intensities ("*x*") were classified into two groups for detailed comparison, then quantified (top right). Quantified data are shown in mean ± s.d. (n = 3 independent experiments). ***, $P < 0.001$ (unpaired *t*-test). CBB, Coomassie Brilliant Blue–stained membrane. **d,** 3D-SIM images acquired from the same samples in (**c**) displaying representative localization patterns for Cep152, Plk4, and Sas6 signals at the centrosome of each cell line. **e,** Overlaid structures of the CPB CP_v1 (brown) and CPB CP (green) showing their overall morphologies. Between the two structures, each PB1 and PB2 domain aligned well with the Cα rmsd values of 0.6970 Å and 0.7679 Å, respectively. **f,** Transmission electron microscopy (TEM) images showing CPB and CPB CP after negative staining. A dimeric, X-shaped morphology is apparent for both proteins. **g,** The $2|F\text{o}|\text{-}|F\text{c}|$ electron density maps are contoured to 1.5 σ around the four residues (S698E S700E, T704E, and T707D) constituting the CP mutations. **h,** Comparison of *B*-factors for the Cα atom of each residue between apo-CPB and CPB CP_v1. The residues with no detectable electron densities are marked with vertical dotted lines (also see the curved dotted lines in (**i**) below). A discontinuity of the *B*-factor profile suggests a disorderliness of the corresponding region [4,5]. Apo-CPB [1] and CPB CP_v1 were refined at 2.60 Å and 2.64 Å, respectively, thus diminishing the likelihood that the variations in *B*-factors are contributed by different structural resolutions. **i,** The overall structures of apo-CPB and CPB CP_v1 show that the PB2-tip region and a broader PB1 wing edge region are disordered (marked by brown dotted lines) in comparison to those in apo-CPB (grey rigid lines). Unlike the PB1 wing edge region, crystal packing did not appear to influence the disordered PB2 tip region. **j,** Identification of a region in CPB that shows potential to become an IDR. Four PONDR predictors revealed probable disordered ("D") residues in the PB2-tip region. Numbers indicate amino acid residues. **k,** Multiple sequence alignment for the PB2-tip region was generated using the Clustal Omega software. The Y750, L752, V758, and L761 residues mutated in the PB2-tip mutant are marked by blue lines. Brown dots indicate residues with no detectable side-chain densities in the crystal structure, while red dots indicate residues whose entire densities were absent from the structure. Hydrophobicity analysis using the ExPASy Kyte-Doolittle method [6] is shown below.

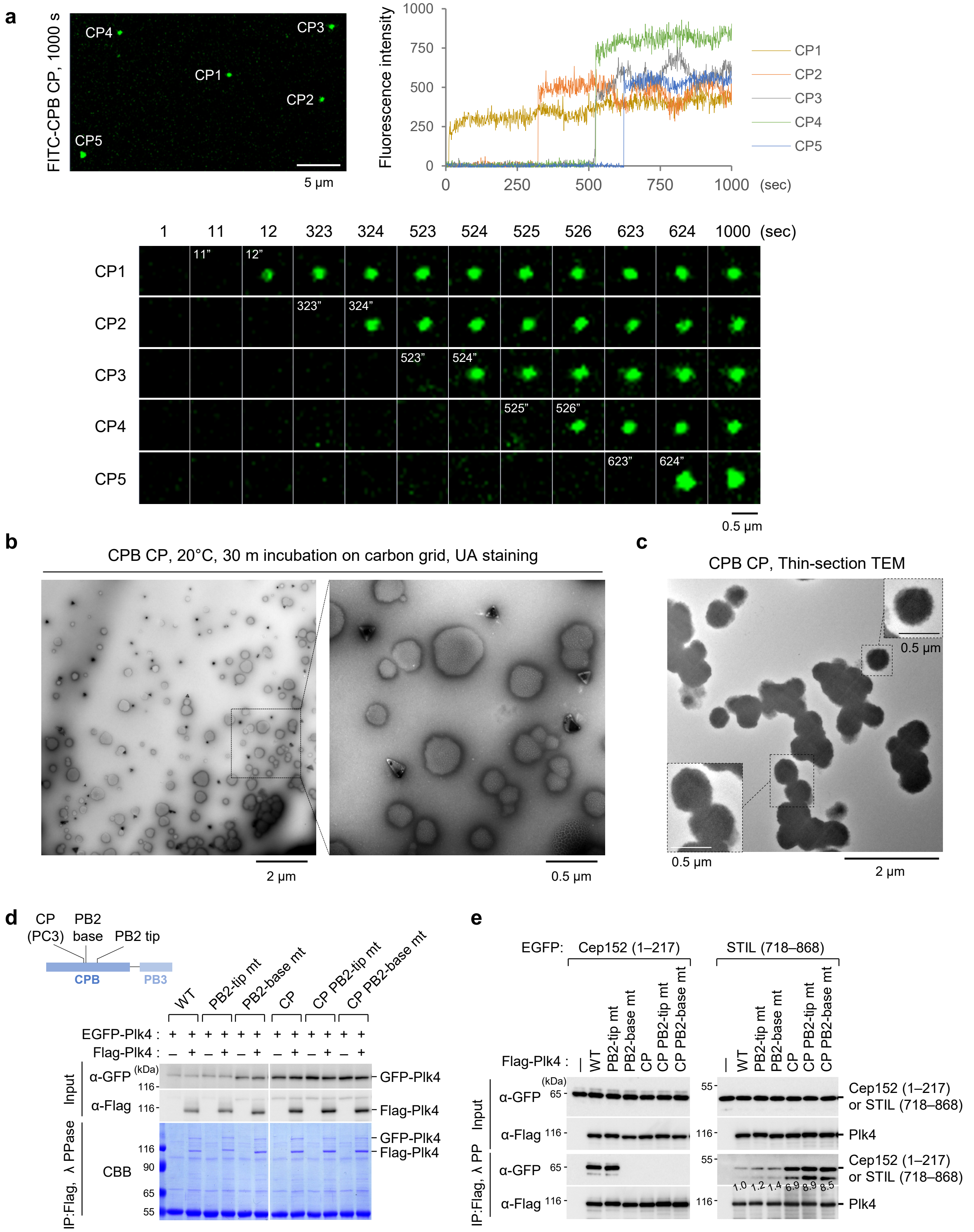
a
FITC-CPB CP, 1000 s
CP4
CP3
CP1
CP2
CP5
5 µm
Fluorescence intensity
1000
750
500
250
0
0
250
500
750
1000
(sec)
CP1
CP2
CP3
CP4
CP5
1 11 12 323 324 523 524 525 526 623 624 1000 (sec)
CP1
CP2
CP3
CP4
CP5
11”
12”
323”
324”
523”
524”
525”
526”
623”
624”
0.5 µm
b
CPB CP, 20°C, 30 m incubation on carbon grid, UA staining
2 µm
0.5 µm
c
CPB CP, Thin-section TEM
0.5 µm
0.5 µm
2 µm
d
CP (PC3)
PB2 base
PB2 tip
CPB
PB3
WT
PB2-tip mt
PB2-base mt
CP
CP PB2-tip mt
CP PB2-base mt
EGFP-Plk4 :
Flag-Plk4 :
Input
α-GFP
α-Flag
(kDa)
116
116
GFP-Plk4
Flag-Plk4
IP:Flag, λ PPase
CBB
116
90
65
55
GFP-Plk4
Flag-Plk4
e
EGFP:
Cep152 (1–217)
STIL (718–868)
Flag-Plk4 :
WT
PB2-tip mt
PB2-base mt
CP
CP PB2-tip mt
CP PB2-base mt
Input
α-GFP
α-Flag
IP:Flag, λ PP
α-GFP
α-Flag
(kDa)
65
116
65
116
55
116
55
116
Cep152 (1–217) or STIL (718–868)
Plk4
Cep152 (1–217) or STIL (718–868)
Plk4
1.0
1.2
1.4
6.9
8.9
8.5

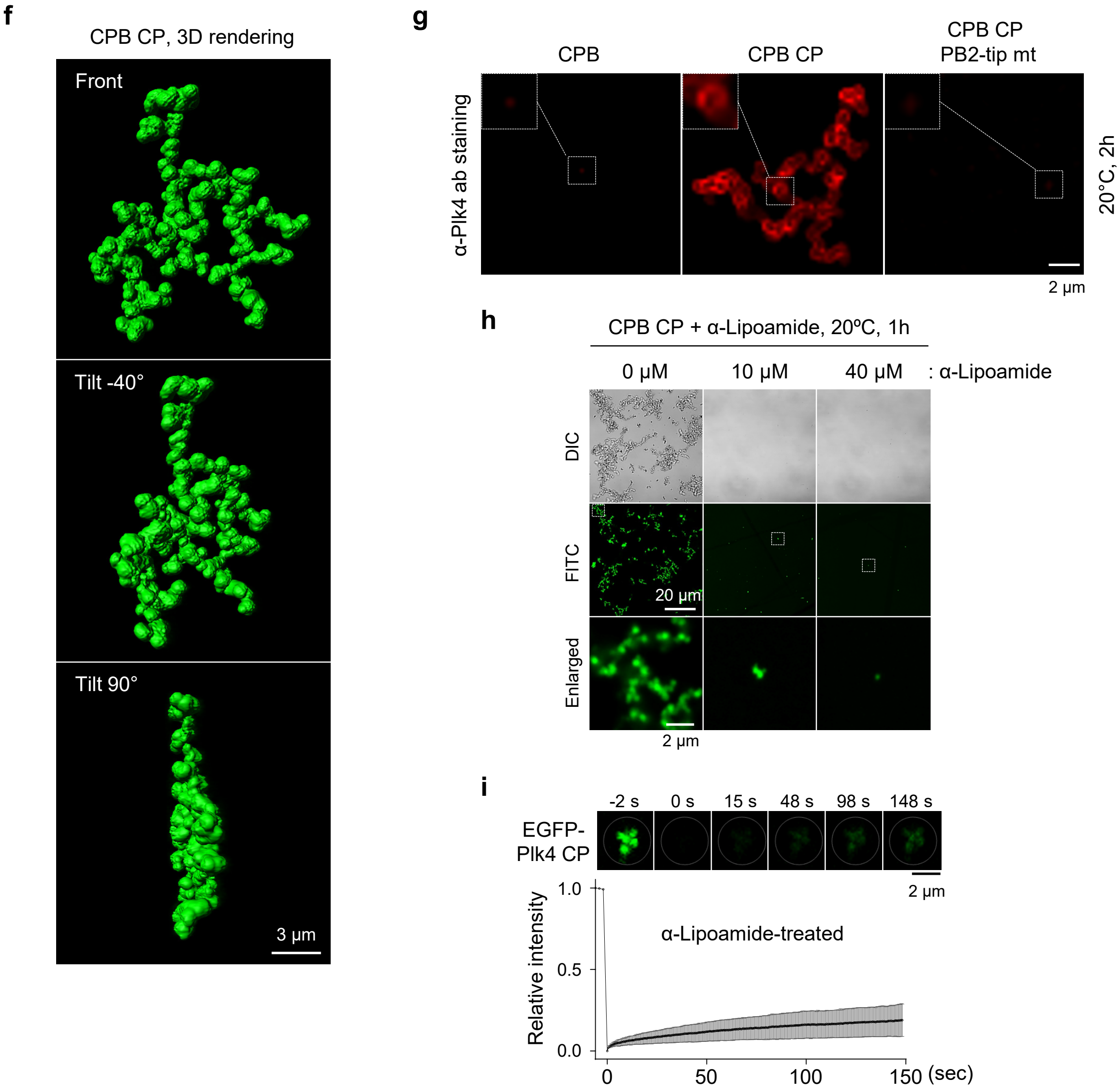

Supplementary Figure 6 | Formation of CPB CP condensates is rapid and sensitive to weak hydrophobic interaction disruptors. a, Time-lapse imaging showing the formation of CPB CP coagulates in real time. Upon incubating FITC–conjugated CPB CP at 20°C, images were acquired at one second intervals. A still image (top left) shows five (CP1–CP5) independent coagulates imaged at the end of experiment. Actual images captured at the indicated time points are shown at the bottom. A graph (top right) shows fluorescence signal intensities for each coagulate quantified as a function of time. Note that coagulates form instantaneously (on the second timescale) independently of one another and their sizes do not appear to change significantly over time. **b,** An example of a transmission electron microscopy (TEM) image showing negatively stained CPB CP coagulates formed *in vitro*. Dotted box, an area of enlargement. **c,** An example of a thin-section TEM image showing no distinguishable structural features inside CPB CP coagulates generated *in vitro*. **d,** IP analyses using HEK293T cells cotransfected with the indicated constructs. Schematic diagram shows the location of PC3, PB2-base, and PB2-tip regions within Plk4 CTD. CBB, Coomassie Brilliant Blue–stained membrane. **e,** IP and immunoblotting analyses using HEK293T cells cotransfected with the indicated constructs. Numbers, relative signal intensities. Note that, unlike the

mutations in the flexible PB2-tip, PB2-base mutations (Y705F, K711A, and N717A) in the structurally well-ordered region (Supplementary Fig. 5i) abnormally disrupted Cep152 binding even in the absence of the CP mutation (lane 4) and therefore they were not included for further investigation. **f,** 3D surface-rendered images of the CPB CP coagulates in Fig. 6d are shown in three different angles. **g,** Confocal analysis after incubating the indicated protein at 20°C for 2 h and stained with anti-Plk4 antibody to visualize the coagulates. Note that due to antibody accessibility, anti-Plk4 signals decorate only the periphery of the coagulates, thus yielding an inside-empty ring morphology. **h,** DIC and confocal analyses after incubating CPB CP at 20°C for 1 h in the presence of the indicated concentrations of α-lipoamide (6,8-dithiooctanoic amide), another small molecule proposed to disrupt protein-driven LLPS [7]. The resulting CPB CP coagulates were visualized by decorating them with FITC. Dotted boxes, areas of enlargement. **i,** Fluorescent recovery after photobleaching (FRAP) analysis was conducted simultaneously with the samples shown in Fig. 6g under the same conditions, except that EGFP-Plk4 CP-expressing U2OS cells were treated with 500 μM (0.01%) of α-lipoamide for 10 min. Images are from Supplementary Video 3. Relative signal intensities were quantified from 16 independent coagulates. Bars, s.d.

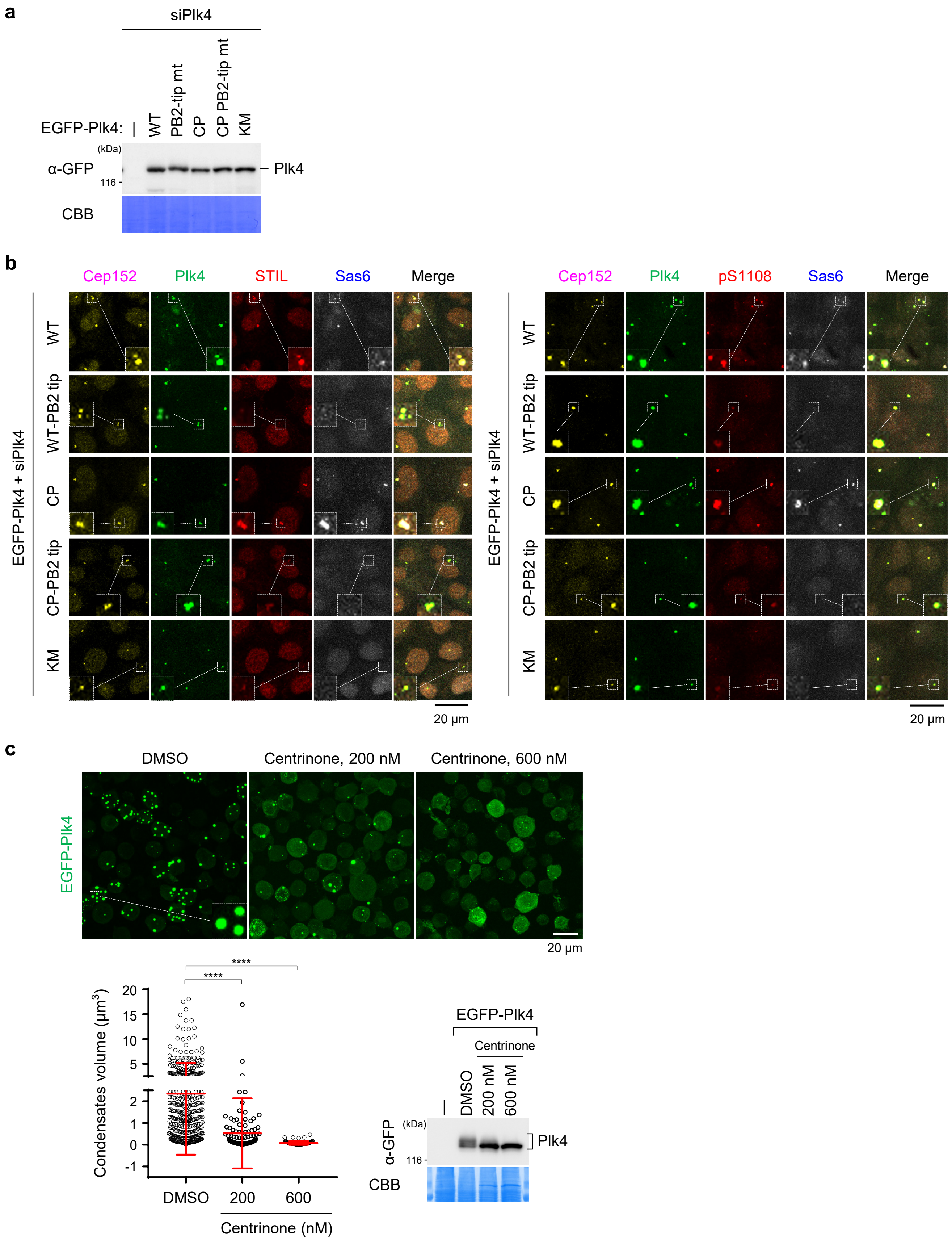

a
siPlk4
EGFP-Plk4:
WT
PB2-tip mt
CP
CP PB2-tip mt
KM
(kDa)
α-GFP
116
Plk4
CBB
b
Cep152
Plk4
STIL
Sas6
Merge
pS1108
WT
WT-PB2 tip
CP
CP-PB2 tip
KM
EGFP-Plk4 + siPlk4
20 µm
c
DMSO
Centrinone, 200 nM
Centrinone, 600 nM
EGFP-Plk4
20 µm
Condensates volume (µm³)
****
DMSO
200
600
Centrinone (nM)
EGFP-Plk4
Centrinone
200 nM
600 nM
(kDa)
α-GFP
116
Plk4
CBB

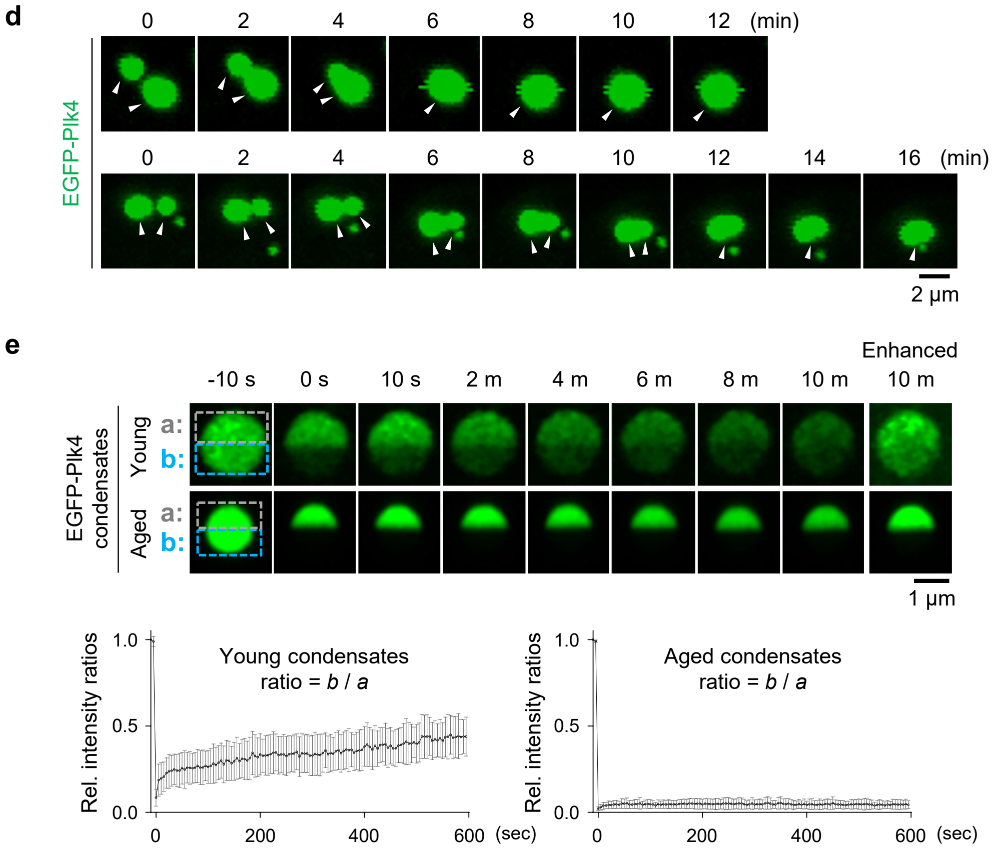

Supplementary Figure 7 | The Plk4 PB2-tip mutant is defective in recruiting STIL and Sas6 to centrosomes. a,b, Immunoblotting and confocal analyses with U2OS cells stably expressing the indicated EGFP-Plk4 constructs under Plk4 RNAi (siPlk4) conditions. Immunoblotting in (**a**) shows the expression levels of the indicated constructs. Dotted boxes in (**b**), areas of enlargement. Quantified data from (**b**) are provided in Fig. 7a. Note that mutations at the PB2 tip disrupted the ability of Plk4 to recruit STIL and Sas6 to centrosomes. **c,** Confocal analysis showing the formation of catalytic activity–dependent, spherical Plk4 condensates in Sf9 cells. Cells expressing EGFP-Plk4 were treated with either control DMSO or the indicated concentration of centrinone for 12 h. The resulting cells were then fixed with 4% paraformaldehyde for 10 min prior to microscopic analysis (top and bottom left) and subjected to immunoblotting analysis (bottom right). Dotted box, an area of image enlargement. The diameter and volume of spherical condensates were quantified. Data are shown in mean ± s.d. (n = 376 for DMSO, 128 for 200 nM, and 94 for 600 nM from 3 independent experiments). ****, $P < 0.0001$ (unpaired *t*-test). CBB, Coomassie Brilliant Blue–stained membrane. Note that the disappearance of spherical Plk4 condensates tightly correlates with the inhibition of Plk4's autophosphorylation activity. **d,** Two representative time-lapse series (out of 14 total) of EGFP-Plk4 condensates in Sf9 cells taken at a 2-min interval. Arrows indicate two spherical condensates undergoing fusion with each other. **e,** FRAP was carried out after photobleaching the bottom half (box "b") of an EGFP-Plk4 condensate purified from Sf9 cells, washed, and placed in a buffer (see details in Methods). Images taken for 10 min are shown along with the image taken before (–10 seconds) photobleaching (top). Relative signal intensities (bottom) were quantified from six young and 12 old photobleached hemispheres ("b") and their corresponding unbleached hemispheres ("a"). Young condensates exhibited distinguishably weaker EGFP fluorescence than the aged ones. Bars, s.d. Dynamic internal rearrangement within a condensate was observed only with young condensates.

Supplementary Figure 8 (Source Data)

Figure 1d

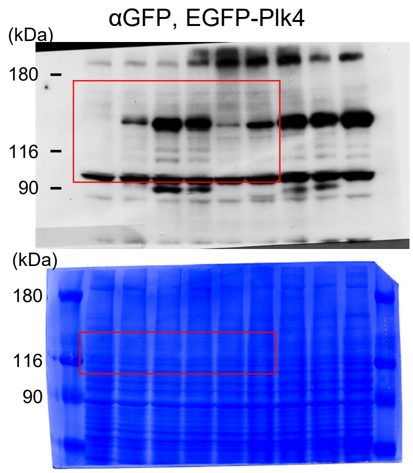

Figure 2d

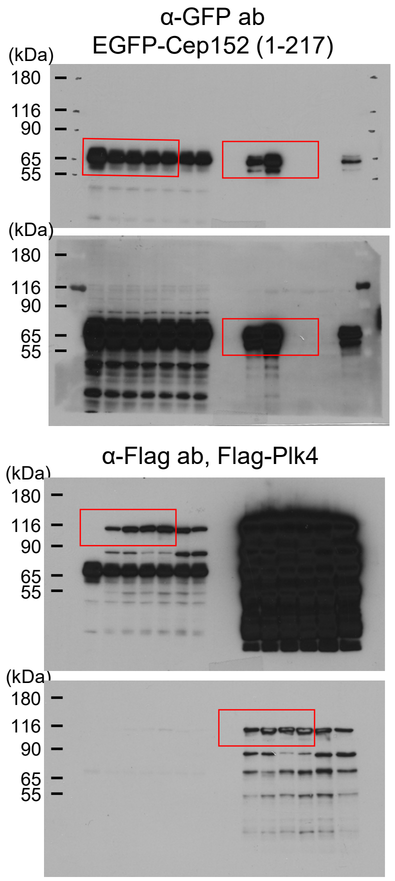

Figure 2e

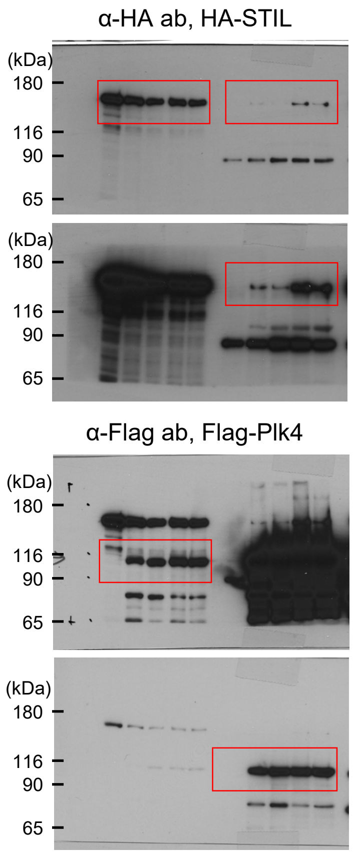

Figure 2f

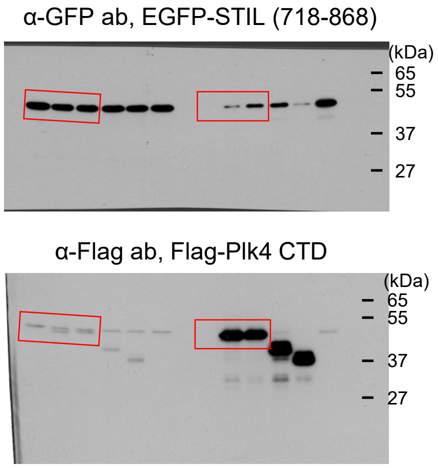

Supplementary Figure 8 (Source Data)

Figure 4a

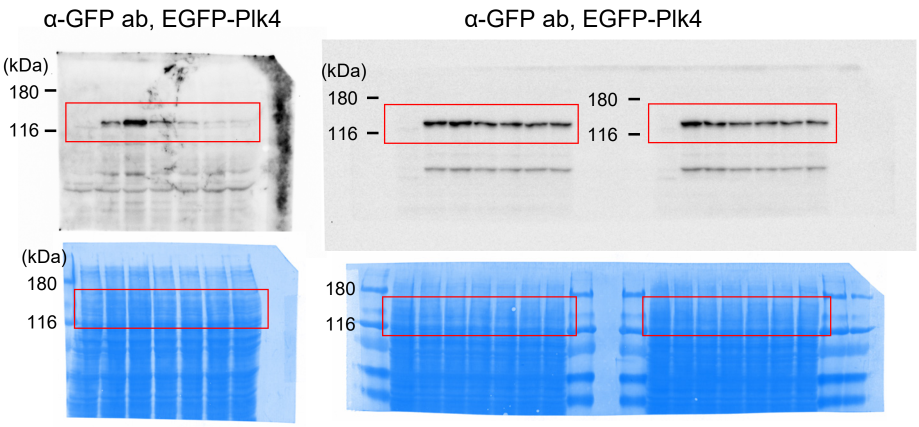

Figure 7a

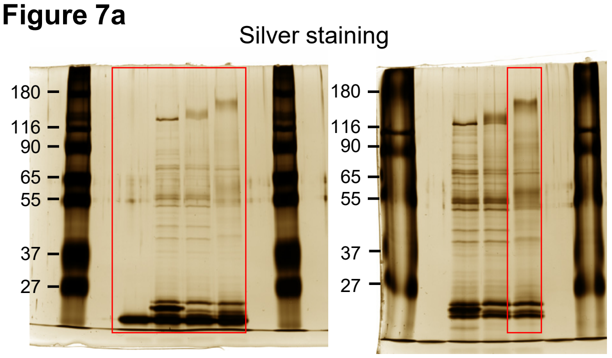

Figure 7b

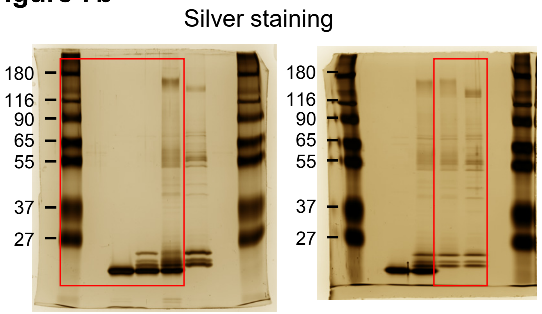

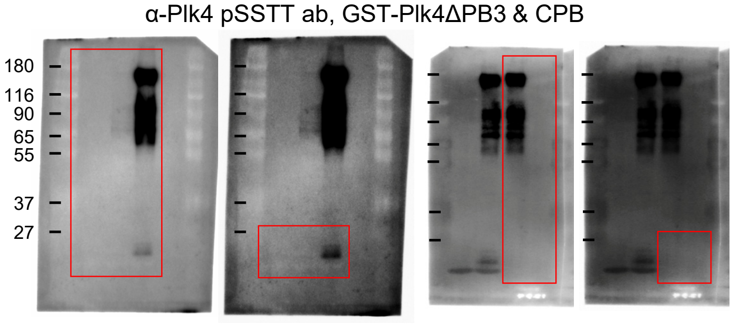

Supplementary Figure 8 (Source Data)

Supplementary Figure 1b

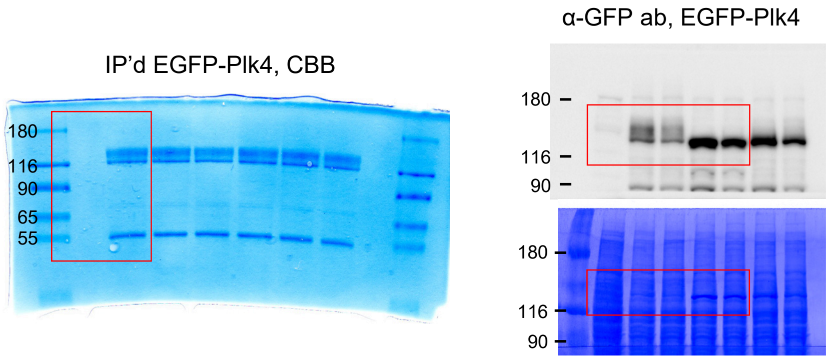

Supplementary Figure 1e

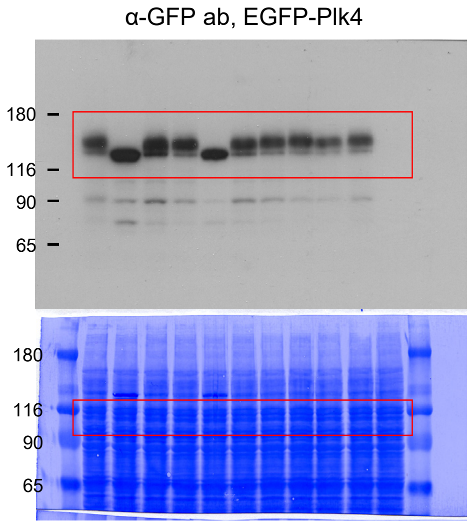

Supplementary Figure 1f

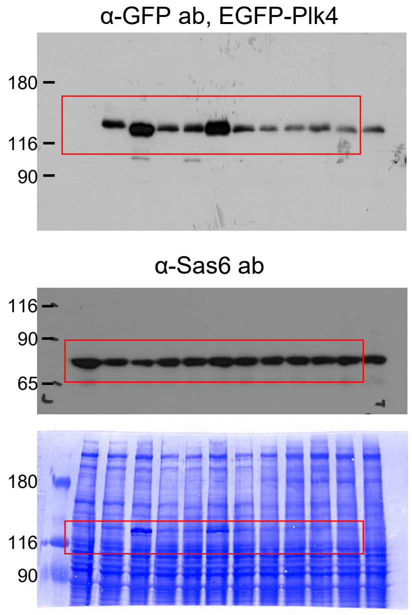

Supplementary Figure 1g

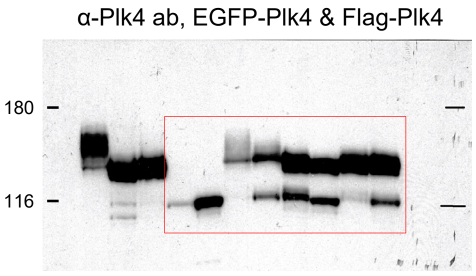

Supplementary Figure 8 (Source Data)

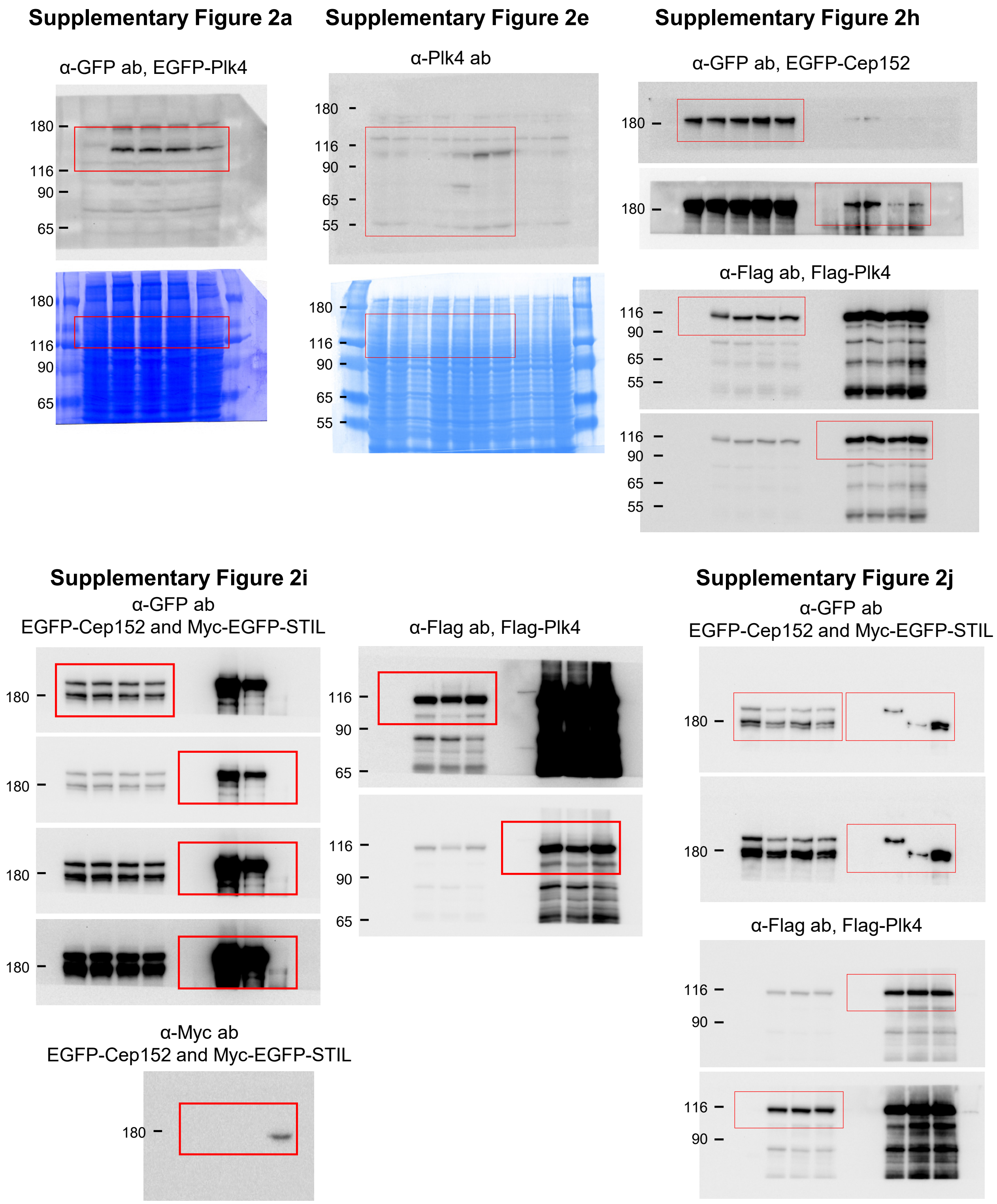

Supplementary Figure 8 (Source Data)

Supplementary Figure 2k

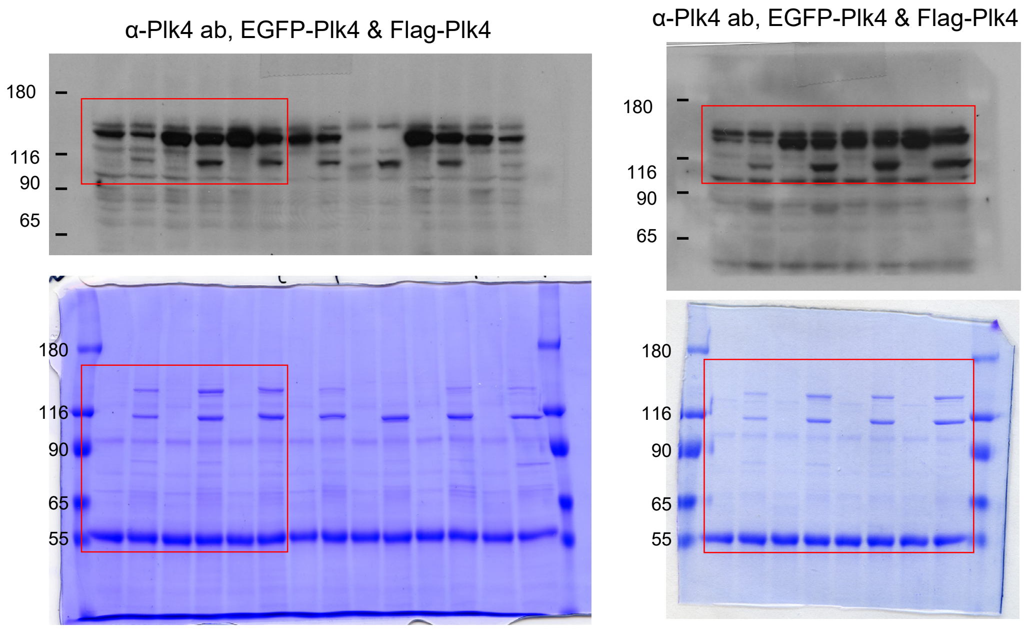

Supplementary Figure 4b

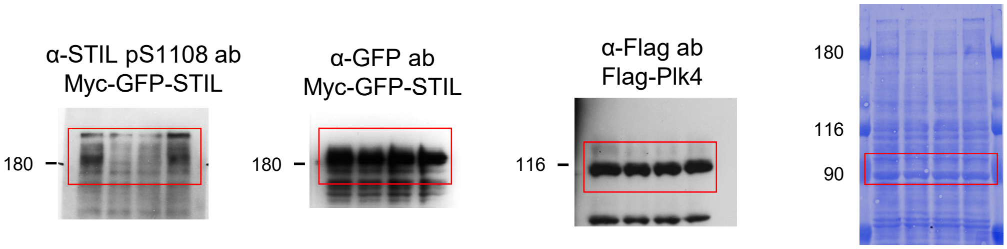

Supplementary Figure 4c

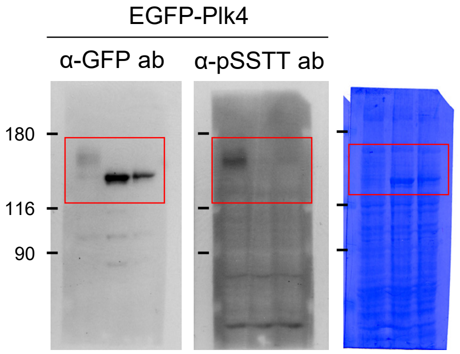

Supplementary Figure 4d

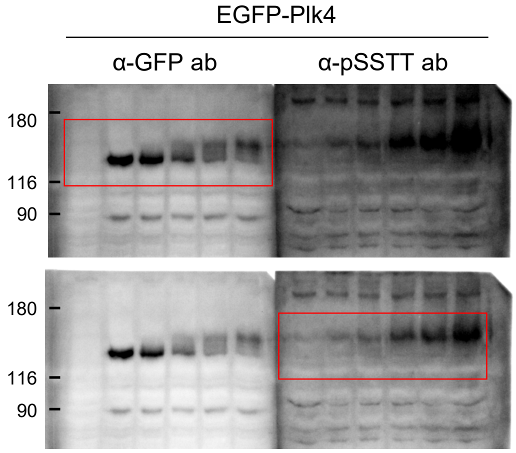

Supplementary Figure 8 (Source Data)

Supplementary Figure 5b

α-GFP ab, EGFP-Cep152

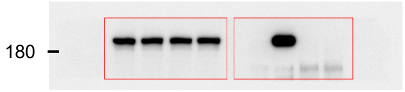

α-Flag ab, Flag-Plk4

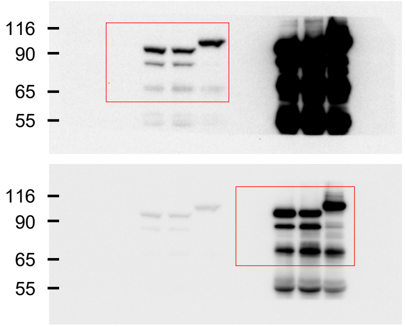

Supplementary Figure 5c

α-GFP ab, EGFP-Plk4

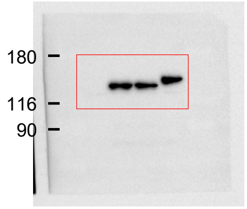

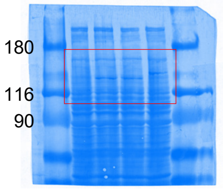

Supplementary Figure 6d

α-GFP ab, EGFP-Plk4 & Flag-Plk4

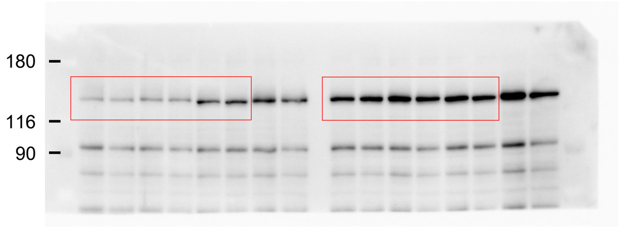

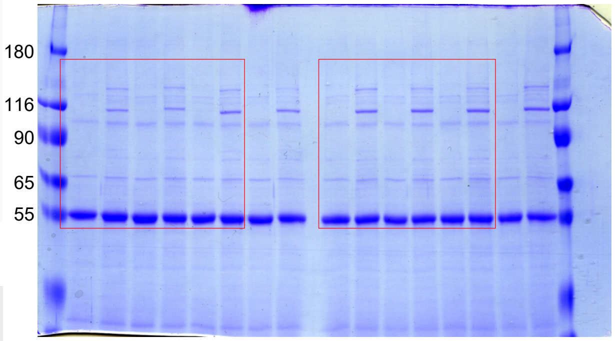

α-Flag ab, EGFP-Plk4 & Flag-Plk4

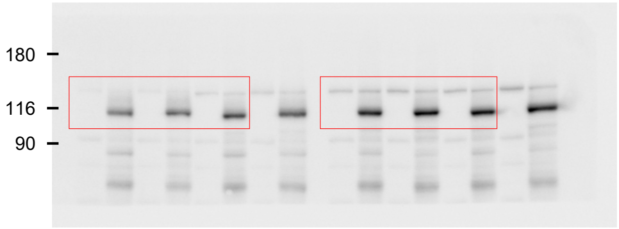

Supplementary Figure 8 (Source Data)

Supplementary Figure 6e

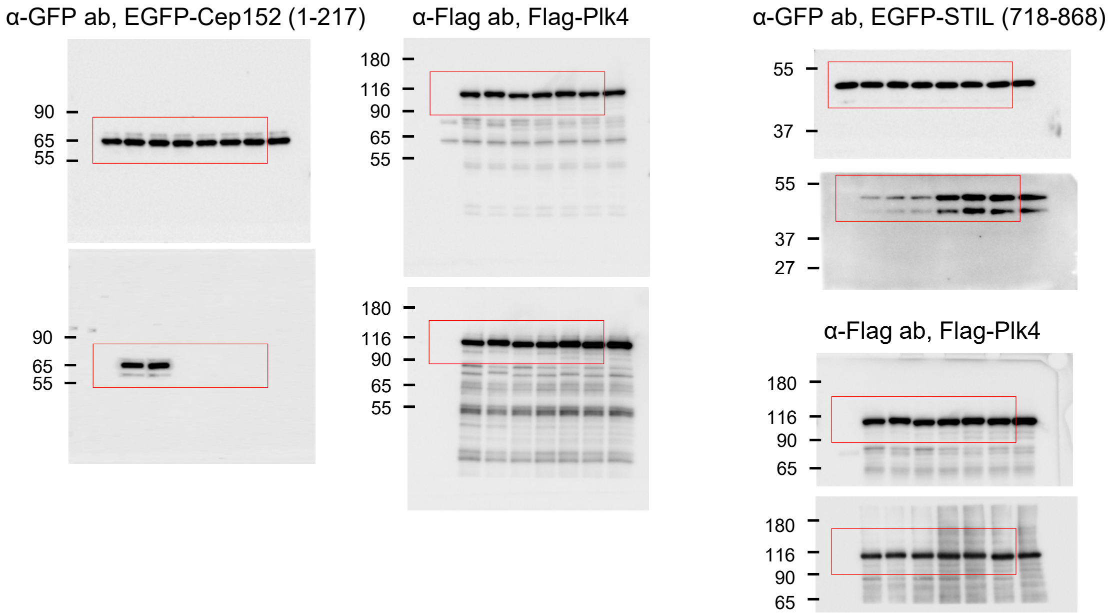

Supplementary Figure 7a

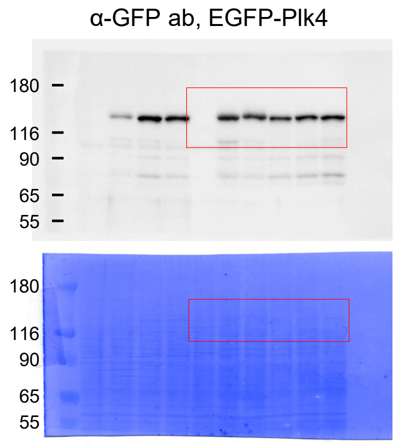

Supplementary Figure 7c

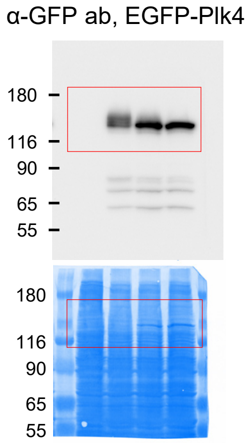

Supplementary Videos

Supplementary Video 1. Time-lapse video of the data shown in Supplementary Fig. 2d.

Supplementary Video 2. 3D reconstruction of images shown in Fig. 3c.

Supplementary Video 3. Time-lapse video of the data shown in Fig. 6g and Supplementary Fig. 6i.

Supplementary References

1 Park, S. Y. *et al.* Molecular basis for unidirectional scaffold switching of human Plk4 in centriole biogenesis. *Nat. Struct. Mol. Biol.* **21**, 696-703, doi:10.1038/nsmb.2846 (2014).

2 Ohta, M. *et al.* Direct interaction of Plk4 with STIL ensures formation of a single procentriole per parental centriole. *Nat. Commun.* **5**, 5267, doi:10.1038/ncomms6267 (2014).

3 Moyer, T. C., Clutario, K. M., Lambrus, B. G., Daggubati, V. & Holland, A. J. Binding of STIL to Plk4 activates kinase activity to promote centriole assembly. *J. Cell Biol.* **209**, 863-878, doi:10.1083/jcb.201502088 (2015).

4 Fenwick, R. B., van den Bedem, H., Fraser, J. S. & Wright, P. E. Integrated description of protein dynamics from room-temperature X-ray crystallography and NMR. *Proc. Natl. Acad. Sci. USA* **111**, E445-454, doi:10.1073/pnas.1323440111 (2014).

5 Hartmann, H. *et al.* Conformational substates in a protein: structure and dynamics of metmyoglobin at 80 K. *Proc. Natl. Acad. Sci. USA* **79**, 4967-4971 (1982).

6 Kyte, J. & Doolittle, R. F. A simple method for displaying the hydropathic character of a protein. *J. Mol. Biol.* **157**, 105-132 (1982).

7 Wheeler, R. J. *et al.* Small molecules for modulating protein driven liquid-liquid phase separation in neurodegenerative disease. *Mol. Biol. Cell* **28**, 3727 (2017).

8 Kim, T.-S. *et al.* Hierarchical recruitment of Plk4 and regulation of centriole biogenesis by two centrosomal scaffolds, Cep192 and Cep152. *Proc. Natl. Acad. Sci. USA* **110**, E4849-4857 (2013).

9 Elbashir, S. M. *et al.* Duplexes of 21-nucleotide RNAs mediate RNA interference in cultured mammalian cells. *Nature* **411**, 494-498. (2001).

10 Cizmecioglu, O. *et al.* Cep152 acts as a scaffold for recruitment of Plk4 and CPAP to the centrosome. *J. Cell Biol.* **191**, 731-739 (2010).

11 Guderian, G., Westendorf, J., Uldschmid, A. & Nigg, E. A. Plk4 trans-autophosphorylation regulates centriole number by controlling betaTrCP-mediated degradation. *J. Cell Sci.* **123**, 2163-2169, doi:10.1242/jcs.068502 (2010).

12 Guardavaccaro, D. *et al.* Control of meiotic and mitotic progression by the F box protein beta-Trcp1 in vivo. *Dev. Cell* **4**, 799-812 (2003).

Supplementary Table 1 | Plasmid constructs used in this study

List	Description	Reference
pKM4489	pHR′.J-CMV-SV-puro/EGFP-Plk4-*sil*	This study
pKM4491	pHR′.J-CMV-SV-puro/EGFP-Plk4 KM-*sil* (K41M)	This study
pKM4691	pHR′.J-CMV-SV-puro/EGFP-Plk4 pc1-*sil* (T586A, S589A, T591A, S592A)	This study
pKM4692	pHR′.J-CMV-SV-puro/EGFP-Plk4 pc2-*sil* (S665A, S671A, S674A)	This study
pKM4693	pHR′.J-CMV-SV-puro/EGFP-Plk4 pc3-*sil* (S698A, S700A, T704A, T707A)	This study
pKM4694	pHR′.J-CMV-SV-puro/EGFP-Plk4 pc4-*sil* (T746A, S749A, T751A, S754A, S756A, S760A)	This study
pKM4696	pHR′.J-CMV-SV-puro/EGFP-Plk4 pc5-*sil* (T793A, S795A)	This study
pKM4697	pHR′.J-CMV-SV-puro/EGFP-Plk4 pc6-*sil* (S809A, T810A, S812A, S817A, S821A)	This study
pKM5205	pHR′.J-CMV-SV-puro/EGFP-Plk4 pc7-*sil* (T864A, T865A, T866A, S868A, T870A, S873A, S874A, S876A)	This study
pKM4699	pHR′.J-CMV-SV-puro/EGFP-Plk4 pc8-*sil* (S956A)	This study
pKM5208	pHR′.J-CMV-SV-puro/EGFP-Plk4 pc1–pc8-*sil* (T586A, S589A, T591A, S592A, S665A, S671A, S674A, S698A, S700A, T704A, T707A, T746A, S749A, T751A, S754A, S756A, S760A, T793A, S795A, S809A, T810A, S812A, S817A, S821A, T864A, T865A, T866A, S868A, T870A, S873A, S874A, S876A, S956A)	This study
pKM5207	pHR′.J-CMV-SV-puro/EGFP-Plk4 pc1–pc8 + PC3-*sil* (T586A, S589A, T591A, S592A, S665A, S671A, S674A, T746A, S749A, T751A, S754A, S756A, S760A, T793A, S795A, S809A, T810A, S812A, S817A, S821A, T864A, T865A, T866A, S868A, T870A, S873A, S874A, S876A, S956A)	This study
pKM4899	pHR′.J-CMV-SV-puro/EGFP-Plk4 CP-*sil* (S698E, S700E, T704E, T707D)	This study
pKM4903	pHR′.J-CMV-SV-puro/EGFP-Plk4 KM CP-*sil* (K41M, S698E, S700E, T704E, T707D)	This study
pKM6562	pHR′.J-CMV-SV-puro/EGFP-Plk4 PB2-tip mutant-*sil* (Y750A, L752A, V758A, L761A)	This study
pKM6564	pHR′.J-CMV-SV-puro/EGFP-Plk4 PB2-base mutant-*sil* (Y705F, K711A, N717A)	This study
pKM6527	pHR′.J-CMV-SV-puro/EGFP-Plk4 CP PB2-tip mutant-*sil* (S698E, S700E, T704E, T707D, Y750A, L752A, V758A, L761A)	This study
pKM6534	pHR′.J-CMV-SV-puro/EGFP-Plk4 CP PB2-base mutant-*sil* (S698E, S700E, T704E, T707D, Y705F, K711A, N717A)	This study

pKM7171	pHR′.J-CMV-SV-puro/EGFP-Plk4 CP_v1-*sil* (S698E, S700E, T704E, T707D)	This study
pKM4530	pHR′.J-CMV-SV-puro/$FLAG_3$-Plk4-*sil*	This study
pKM4532	pHR′.J-CMV-SV-puro/$FLAG_3$-Plk4 KM-*sil* (K41M)	This study
pKM4703	pHR′.J-CMV-SV-puro/$FLAG_3$-Plk4 pc3-*sil* (S698A, S700A, T704A, T707A)	This study
pKM5070	pHR′.J-CMV-SV-puro/$FLAG_3$-Plk4 CP-*sil* (S698E, S700E, T704E, T707D)	This study
pKM5071	pHR′.J-CMV-SV-puro/$FLAG_3$-Plk4 KM CP-*sil* (K41M, S698E, S700E, T704E, T707D)	This study
pKM6565	pHR′.J-CMV-SV-puro/$FLAG_3$-Plk4 PB2-tip mutant-*sil* (Y750A, L752A, V758A, L761A)	This study
pKM6567	pHR′.J-CMV-SV-puro/$FLAG_3$-Plk4 PB2-base mutant-*sil* (Y705F, K711A, N717A)	This study
pKM6559	pHR′.J-CMV-SV-puro/$FLAG_3$-Plk4 CP PB2-tip mutant-*sil* (S698E, S700E, T704E, T707D, Y750A, L752A, V758A, L761A)	This study
pKM6561	pHR′.J-CMV-SV-puro/$FLAG_3$-Plk4 CP PB2-base mutant-*sil* (S698E, S700E, T704E, T707D, Y705F, K711A, N717A)	This study
pKM4591	pHR′.J-CMV-SV-puro/$FLAG_3$-Plk4 CTD-*sil*	This study
pKM5764	pHR′.J-CMV-SV-puro/$FLAG_3$-Plk4 CP CTD-*sil*	This study
pKM4483	pCI-neo/HA-STIL	This study
pKM5489	pHR′.J-CMV-SV-puro/EGFP-STIL coiled coil [718-868]	This study
pKM7162	pHR′.J-Endo-SV-puro/Plk4-*sil*	This study
pKM7164	pHR′.J-Endo-SV-puro/Plk4 CP-*sil*	This study
pKM7167	pHR′.J-Endo-SV-puro/Plk4 CP PB2-tip mt-*sil*	This study
pKM6886	pHR′.J-CMV-SV-puro/$FLAG_3$-Plk4 [1-808]	This study
pKM6887	pHR′.J-CMV-SV-puro/$FLAG_3$-Plk4 CP [1-808] (S698E, S700E, T704E, T707D)	This study
pKM6889	pHR′.J-CMV-SV-puro/$FLAG_3$-Plk4 CP [1-808]_v1 (S698E, S700E, T704E, T707D)	This study
pKM3686	His_6-MBP-TEV-CPB	[1]
pKM5401	His_6-CPB CP (S698E, S700E, T704E, T707D)	This study
pKM6515	His_6-MBP-TEV-CPB CP (S698E, S700E, T704E, T707D)	This study
pKM6892	His_6-MBP-TEV-CPB CP PB2-tip mutant	This study
pKM5850	His_6-MBP-TEV-CPB CP_v1 (S698E, S700E, T704E, T707D)	This study
pKM3561	pEGFPc1-Cep152 (1-217aa)	[8]
pKM3841	pEGFPc1-Cep152	[8]
pKM4572	pDEST-N-FLAG-βTrCP2	Lab stock

pKM6348	pCDNA5/FRT/TO/Myc-GFP-STIL[Full length]	[3]
pKM6349	pCDNA5/FRT/TO/Myc-GFP-STIL[S1108A]	[3]
pKM6350	pCDNA5/FRT/TO/Myc-GFP-STIL[S1116A]	[3]
pKM6351	pCDNA5/FRT/TO/Myc-GFP-STIL[S1108A,S1116A]	[3]
pKM4884	pAcJ-EGFP-Plk4-*sil*	This study

Supplementary Table 2 | siRNA sequences used in this study

Target Gene	Sequence (nt positions from the start codon)	Reference
Luciferase	CGTACGCGGAATACTTCGA	[9]
Cep152	GCGGATCCAACTGGAAATCTA (3099–3119)	[10]
Plk4	AAGGACTTGGTCTTACAACTAdTdT (2576–2596)	This study
Plk4 (3'UTR)	CTCCTTTCAGACATATAAG	[11]
STIL	GCTCCAAACAGTTTCTGCTGGAAT (1611–1633)	[3]
βTrCP1/2	GTGGAATTTGTGGAACATCTTdTdT	[12]

Supplementary Table 3 | Antibodies used in this study

Antibody	Source	Cat #
Rabbit anti-Plk4 (580-970)	[8]	N/A
Mouse anti-Plk4 clone 6H5	Millipore	MABC544
Rabbit anti-Cep152 (491-810)	[8]	N/A
Mouse anti-Sas6 (404-657)	Santa Cruz Biotech	sc-81431
Rabbit anti-GFP	Santa Cruz Biotech	sc-8334
Rat anti-GFP	MBL international	D153-3
Mouse anti-GFP	Santa Cruz Biotech	sc-9996
Rabbit anti-STIL (1237-1287)	Abcam	ab89314
Alexa Fluor 647–conjugated anti-Cep152 (491-810)	This study	N/A
Mouse anti-FLAG M2-Peroxidase (HRP) antibody	SIGMA	A8592
Mouse anti-FLAG (clone M2)	Sigma	F1804
Mouse anti-FLAG M2 Affinity Gel	SIGMA	A2220
Rat anti-HA (clone 3F10)	Roche	11867423001
Rabbit anti-Cyclin A	Santa Cruz Biotech	sc-751
Rabbit anti-GFP	Abcam	ab6556
Mouse anti-Acetylated tubulin	Sigma	T7451
Rabbit anti-Plk4 pSSTT	This study	N/A
Alexa Fluor 594–conjugated anti-Plk4 pSSTT	This study	N/A
Rabbit anti-STIL pS1108	This study	N/A
Mouse Anti-Myc	Lab stock	N/A
Goat-anti-rabbit IgG; EM-grade6 nm	Electron Microscopy Sciences (EMS)	25104
Alexa Fluor 594 Donkey anti-mouse IgG(H+L)	Thermo Fisher Scientific	A21203
Alexa Fluor 594 Donkey anti-rabbit IgG(H+L)	Thermo Fisher Scientific	A21207
Alexa Fluor 647 Donkey anti-mouse IgG(H+L)	Thermo Fisher Scientific	A31571
Alexa Fluor 647 Donkey anti-rabbit IgG(H+L)	Thermo Fisher Scientific	A31573
Alexa Fluor 405 Goat anti-mouse IgG(H+L)	Thermo Fisher Scientific	A31553
Alexa Fluor 405 Goat anti-rabbit IgG(H+L)	Thermo Fisher Scientific	A31556
Anti-mouse IgG, HRP	GE Healthcare	NA9310V
Anti-rabbit IgG, HRP	GE Healthcare	NA9340V

Supplementary Table 4 | Peptide sequences used in this study

List	Sequence*	Source
pSSTT	CVQLVR**pS**K**pS**PKI**pT**YF**pT**RYAK	This study
SSTT	CVQLVR**S**K**S**PKI**T**YF**T**RYAK	This study
pS1108	CDRSTVGL**pS**LISPN	This study
S1108	CDRSTVGL**S**LISPN	This study

*Phosphorylated residues are marked in boldface type.

Source data file

All numerical source data are provided as a Source Data file. All original uncropped blots and gels are provided with size markers in Supplementary Fig. 8. Cropped regions are marked.
