# Supplementary material for: Phase separation of Polo-like kinase 4 by autoactivation and clustering drives centriole biogenesis
Source: Nat Commun. 2019 Oct 31;10:4959. doi: 10.1038/s41467-019-12619-2 (PMC6823436; doi:10.1038/s41467-019-12619-2)
Supplement: Supplementary file 3 — Description of Additional Supplementary Files [file 41467_2019_12619_MOESM3_ESM.docx]

**Description of Additional Supplementary Files**

**File Name: Supplementary Movie 1**

**Description:** Time-lapse video of the data shown in Supplementary Fig. 2d.

**File Name: Supplementary Movie 2**

**Description:** 3D reconstruction of images shown in Fig. 3c.

**File Name: Supplementary Movie 3**

**Description:** Time-lapse video of the data shown in Fig. 6g and Supplementary

Fig. 6i.
