# Supplementary material for: Phase separation of Polo-like kinase 4 by autoactivation and clustering drives centriole biogenesis
Source: Nat Commun. 2019 Oct 31;10:4959. doi: 10.1038/s41467-019-12619-2 (PMC6823436; doi:10.1038/s41467-019-12619-2)
Supplement: Supplementary file 7 — Reporting Summary [file 41467_2019_12619_MOESM7_ESM.pdf]

nature research

Corresponding author(s): Kyung S. Lee

Last updated by author(s): May 14, 2019

Reporting Summary

Nature Research wishes to improve the reproducibility of the work that we publish. This form provides structure for consistency and transparency in reporting. For further information on Nature Research policies, see Authors & Referees and the Editorial Policy Checklist .

Statistics

For all statistical analyses, confirm that the following items are present in the figure legend, table legend, main text, or Methods section.

n/a	Confirmed	
☐	☒	The exact sample size (*n*) for each experimental group/condition, given as a discrete number and unit of measurement
☐	☒	A statement on whether measurements were taken from distinct samples or whether the same sample was measured repeatedly
☐	☒	The statistical test(s) used AND whether they are one- or two-sided<br>*Only common tests should be described solely by name; describe more complex techniques in the Methods section.*
☐	☒	A description of all covariates tested
☐	☒	A description of any assumptions or corrections, such as tests of normality and adjustment for multiple comparisons
☐	☒	A full description of the statistical parameters including central tendency (e.g. means) or other basic estimates (e.g. regression coefficient) AND variation (e.g. standard deviation) or associated estimates of uncertainty (e.g. confidence intervals)
☐	☒	For null hypothesis testing, the test statistic (e.g. *F*, *t*, *r*) with confidence intervals, effect sizes, degrees of freedom and *P* value noted<br>*Give P values as exact values whenever suitable.*
☒	☐	For Bayesian analysis, information on the choice of priors and Markov chain Monte Carlo settings
☒	☐	For hierarchical and complex designs, identification of the appropriate level for tests and full reporting of outcomes
☒	☐	Estimates of effect sizes (e.g. Cohen's *d*, Pearson's *r*), indicating how they were calculated

Our web collection on statistics for biologists contains articles on many of the points above.

Software and code

Policy information about availability of computer code

Data collection	LSM780 ELIRA S.1 (Zeiss, Germany), LSM880 Airyscan (Zeiss, Germany), H-7650 transmission electron microscope (Hitachi , Japan), SoftWoRx 6.5.2 (GE Healthcare, USA), 22-ID beamline of the Advanced Photon Source (Argonne, IL, USA), EnSpire Multimode Plate reader (PerkinElmer, USA), DU 800 spectrophotometer (Beckman Coulter, USA)
Data analysis	Zen software (Zeiss), Image J (Fiji), GraphPad Prism7, Imaris 8.4.1, Image Lab, Unicorn 7, HKL2000, CCP4 Software Suite 7.0.065, Phenix 1.14-3260, Pymol 2.2.0

For manuscripts utilizing custom algorithms or software that are central to the research but not yet described in published literature, software must be made available to editors/reviewers. We strongly encourage code deposition in a community repository (e.g. GitHub). See the Nature Research guidelines for submitting code & software for further information.

Data

Policy information about availability of data

All manuscripts must include a data availability statement. This statement should provide the following information, where applicable:

- Accession codes, unique identifiers, or web links for publicly available datasets
- A list of figures that have associated raw data
- A description of any restrictions on data availability

The PDB accession codes for the two crystal structures in the manuscript are 6N45 and 6N46 (PDB validation sheets are attached). Raw data are available upon request.

Field-specific reporting

Please select the one below that is the best fit for your research. If you are not sure, read the appropriate sections before making your selection.

- [x] Life sciences
- [ ] Behavioural & social sciences
- [ ] Ecological, evolutionary & environmental sciences

For a reference copy of the document with all sections, see nature.com/documents/nr-reporting-summary-flat.pdf

Life sciences study design

All studies must disclose on these points even when the disclosure is negative.

Sample size	The sample size was determined based on the size of the standard deviation and the reproducibility of the data. For statistical significance, at least three independent experiments were subjected.
Data exclusions	No data were excluded.
Replication	All experiments were successfully reproduced at least three times. The results reliably support conclusions stated in the manuscript.
Randomization	For X-ray crystal structure refinement and model validation, data were randomly split into two groups, one group was used for refinement and the other for validation. For quantification of samples, images were taken randomly from undefined areas. For FRAP experiment, coagulates from in vivo and in vitro experiment were randomly selected for analysis. For quantification of stained samples, images were taken randomly from undefined areas. Randomization of animals or participants is not relevant to this study.
Blinding	Acquisition of images and quantifications were conducted in a blinded manner.

Reporting for specific materials, systems and methods

We require information from authors about some types of materials, experimental systems and methods used in many studies. Here, indicate whether each material, system or method listed is relevant to your study. If you are not sure if a list item applies to your research, read the appropriate section before selecting a response.

Materials & experimental systems

n/a	Involved in the study	
	x	Antibodies
	x	Eukaryotic cell lines
x		Palaeontology
x		Animals and other organisms
x		Human research participants
x		Clinical data

Methods

n/a	Involved in the study	
x		ChIP-seq
x		Flow cytometry
x		MRI-based neuroimaging

Antibodies

Antibodies used	All the antibodies used in this study are listed in the Supplementary Table 4 with Source information, catalog number. Previously published antibodies are referenced. All antibodies are available upon request.
Validation	Antibodies used for immunoprecipitation and immunoblotting (FLAG, HA, and GFP) were internally controlled within our results. Phospho-antibodies for Plk4 pSSTT and STIL pS1108 were validated by immunoblotting with the respective mutant proteins. Antibodies used for immunofluorescence (Plk4, Plk4 pSSTT, Cep152, STIL, STIL pS1108, Sas6, and Cyclin A) were either internally controlled or validated by siRNA. Commercially available antibodies were used according to manufacturer's recommendations.

Eukaryotic cell lines

Policy information about cell lines

Cell line source(s)	U2OS and HEK293 cells were purchased from American Type Culture Collection (ATCC) and cultured as recommended by ATCC.
Authentication	Cells have been authenticated by the vendor and used as low passage cell lines for experiments. No further authentication was performed for cell lines from ATCC.
Mycoplasma contamination	Mycoplasma contamination was tested and found negative.

Commonly misidentified lines
(See ICLAC register)

No commonly misidentified cell lines were used.
